# Adaptive genetic variation to drought in a widely distributed conifer suggests a potential for increasing forest resilience in a drying climate

**DOI:** 10.1111/nph.16551

**Published:** 2020-05-12

**Authors:** Claire Depardieu, Martin P. Girardin, Simon Nadeau, Patrick Lenz, Jean Bousquet, Nathalie Isabel

**Affiliations:** ^1^ Natural Resources Canada Canadian Forest Service Laurentian Forestry Centre 1055, rue du PEPS, PO Box 10380, Stn. Sainte‐Foy Québec QC G1V 4C7 Canada; ^2^ Canada Research Chair in Forest Genomics Institute for Systems and Integrative Biology Université Laval Québec QC G1V 0A6 Canada; ^3^ Natural Resources Canada Canadian Forest Service Canadian Wood Fibre Centre 1055, rue du PEPS, PO Box 10380, Stn. Sainte‐Foy Québec QC G1V 4C7 Canada

**Keywords:** common garden experiment, dendroecology, genetics, local adaptation, provenance trial, tree rings, white spruce

## Abstract

- Drought intensity and frequency are increasing under global warming, with soil water availability now being a major factor limiting tree growth in circumboreal forests. Still, the adaptive capacity of trees in the face of future climatic regimes remains poorly documented.
- Using 1481 annually resolved tree‐ring series from 29‐yr‐old trees, we evaluated the drought sensitivity of 43 white spruce (*Picea glauca* (Moench) Voss) populations established in a common garden experiment.
- We show that genetic variation among populations in response to drought plays a significant role in growth resilience. Local genetic adaptation allowed populations from drier geographical origins to grow better, as indicated by higher resilience to extreme drought events, compared with populations from more humid geographical origins. The substantial genetic variation found for growth resilience highlights the possibility of selecting for drought resilience in boreal conifers.
- As a major research outcome, we showed that adaptive genetic variation in response to changing local conditions can shape drought vulnerability at the intraspecific level. Our findings have wide implications for forest ecosystem management and conservation.

## Introduction

The rising frequency and severity of regional droughts brought on by a warming‐induced reduction in soil water content will negatively affect the growth of boreal tree species and reduce the productivity and the carbon (C) uptake capacity of circumboreal forests (Girardin *et al.*, 2016; Buermann *et al.*, 2018; Reich *et al.*, 2018). The shift detected in baseline climate over the 20^th^ century has already triggered increased impacts of water availability on tree growth (Babst *et al.*, 2019). Questions are also being raised regarding the resilience of forests and tree species in response to expected increases in the frequency and severity of climate disturbances such as droughts and heat waves (D’Orangeville *et al.*, 2018; Wiley *et al.*, 2018; Giguère‐Croteau *et al.*, 2019). Even more worrying is the possibility that negative effects will be amplified through loss of tree vigour and heightened susceptibility to pathogens, insects, and other disturbance agents (Chen *et al.*, 2017; De Grandpré *et al.*, 2019).

Given the rapidity of climatic changes relative to the slow pace of evolutionary changes in tree species, there is an urgent need to assess their adaptive capacity to further predict and help maintain forest productivity under future climate regimes (Aitken *et al.*, 2008). The difficulty of predicting the adaptive capacity of a species is further exacerbated by the complexity and types of functional traits (stomatal conductance, leaf area, water use efficiency, etc.) that may be involved (Aubin *et al.*, 2016). Whereas research emphasizes characterizing the interspecific variation of functional traits, these may also vary within species in response to climate change, which is generally less understood (reviewed by Moran *et al.*, 2017). Indeed, predictive models of tree growth and species dispersal used in impact studies apply functional traits uniformly across populations of a given species in a static manner (Girardin *et al.*, 2016; D’Orangeville *et al.*, 2018). Conversely, population differentiation in functional traits hints at substantial local adaptation to climate (Andalo *et al.*, 2005). Therefore, a better understanding of the interplay between tree adaptation to severe climate disturbances and genetic variation, and their impacts on growth and productivity, should provide valuable insights for predictive modelling and forest management.

Common garden experiments (CGEs) can unravel the genetic basis of complex traits across various provenances and test for signals of local adaptation in life history traits (De Villemereuil *et al.*, 2016). These experiments are generally based on collecting seeds from populations of different geographic origins, called provenances, whose seedlings are planted at a common site. During the 1960s and 1970s, several CGEs were established in eastern Canada with the aim to identify the best seed sources for reforestation of several conifer species. Hence, in white spruce (*Picea glauca* (Moench) Voss), early analyses of provenance trials reported on the geographic differentiation of juvenile growth and winter desiccation (Corriveau & Boudoux, 1971), tree growth and phenology (Li *et al.*, 1997), and wood density (WD) and production (Beaulieu & Corriveau, 1985). More recent studies using these legacy CGEs also allowed for the identification of genetic markers putatively involved in adaptive population differentiation (Namroud *et al.*, 2008) or presented genomic approaches to hasten breeding for complex traits such as growth and wood quality (Beaulieu *et al.*, 2014; Lenz *et al.*, 2020).

**Fig. 1:**
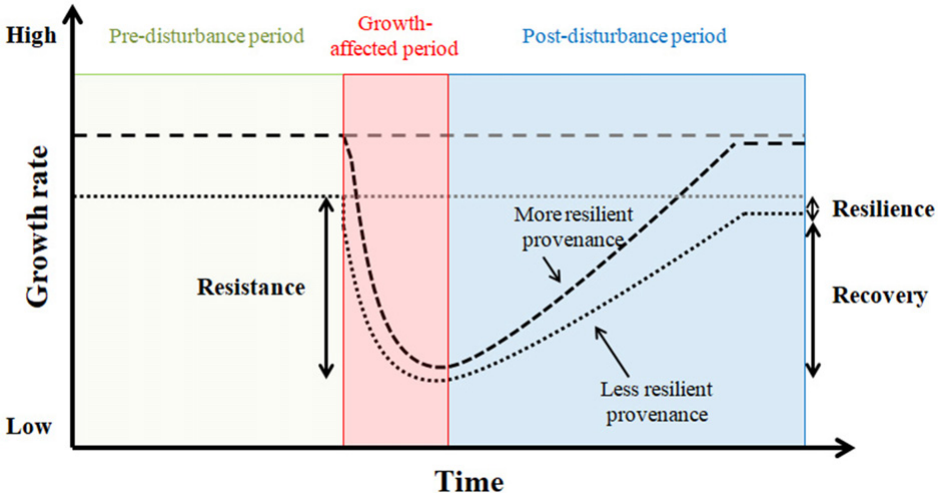
Conceptual diagram of growth resilience. Definition of drought‐resilience traits: resistance, resilience and recovery of radial tree growth increment in response to a drought episode, as used in the present study and as previously defined by Lloret *et al. *(2011).

A CGE arranged in an experimental design such as a randomized complete block design allows for factoring out environmental influences on trait variation and for characterizing the genetic basis of adaptive differentiation in simple dendrometric traits, such as tree height or diameter (Andalo *et al.*, 2005). An emerging opportunity in recent years has been the study of annual growth rings in trees (i.e. dendroecology), in combination with CGEs, to provide retrospective insights as to how trees react to extreme climate events, such as droughts, throughout their lifespan and across populations (Montwé *et al.*, 2016; Housset *et al.*, 2018). Evaluations such as those of climate–growth relationships at a yearly resolution allow the assessment of variation in drought vulnerability within tree species and of local genetic adaptation in an era of rapid climate change (George *et al.*, 2017; Housset *et al.*, 2018). One of the interesting features of this approach is the possibility of studying the ability of trees to continue growing under drought conditions (i.e. resistance) and to recover following a drought stress event (i.e. resilience), observations that cannot be made using conventional studies of dendrometric traits alone (George *et al.*, 2017; see Fig. 1). Though tree‐ring width data offer a means of tracking growth and drought episodes over many years, tracheid traits can more substantially reflect a climate signal and allow the assessment of the hydraulic function of the xylem (Fonti *et al.*, 2010). Specifically, the tracheid diameter is an indicator of drought sensitivity (i.e. the greater the size, the higher the vulnerability), and WD may be used as an indicator of resistance to embolism (i.e. the greater the density, the higher the resistance; Hacke *et al.*, 2001). In spite of a plant’s ability to capture the impacts of drought stress on xylem function, the intraspecific variation of these traits remains poorly documented (Aubin *et al.*, 2016).

In this paper, we provide evidence in support of the hypothesis that variations in drought response play a significant role in local adaptation and in the growth resilience of a widely distributed boreal conifer. To illustrate this, we took advantage of a large tree‐ring database from a CGE to evaluate the adaptive response to drought of 43 white spruce populations. With white spruce being a widespread drought‐sensitive tree species of major ecological and economic importance in North America (Barber *et al.*, 2000; Abrahamson, 2015; Canadian Forest Service, 2015), the assessment of its adaptive capacity to future climates is of particular relevance. In this study, we determined how drought affected radial growth and tracheid traits using a retrospective study of annual resolved tree‐ring series. We further evaluated the growth resilience of trees in response to an extreme drought episode in 2001–2002, the most severe dry spell impacting northeastern boreal forests with conditions not seen for at least 100 yr in some regions (Wheaton *et al.*, 2008). Using quantitative genetics approaches, we also investigated the among and within white spruce provenance intraspecific variation of the tested xylem traits.

## Materials and Methods

### Study site and plant material

The CGE used in this study was established by the Canadian Forest Service in 1979 in Mastigouche, Quebec, Canada (lat 46°38′N, long 73°13′W, elevation 230 m), and is located within the natural distribution of white spruce (Supporting Information Fig. S1). We sampled 1481 trees from 43 white spruce seed sources (referred to as provenances) that are distributed along mean annual precipitation (MAP; 841–1359 mm) and mean annual temperature (MAT; −1.1 to 5.6°C; Table S1) gradients in the province of Quebec, Canada. Provenances were each represented by one to five half‐sib families originating from open pollination, for a total of 197 families. The experimental design consisted of a randomized complete block design. Irrespective of its provenance, each family was randomly repeated by a five‐tree row‐plot in each of the six blocks. The trial was established in 1979 using 4‐yr‐old seedlings planted at a 1.2 m spacing with 2.4 m between each family row.

### Climate variables

Monthly means of daily climate data (maximum, minimum and mean temperature), precipitation (sum, millimetres), relative humidity (per cent), and vapour pressure deficits (kilopascals) for the 43 provenance origins and the common garden site were obtained using the biosim software v.11.4 (Régnière *et al.*, 2017). As part of the procedure, daily data were interpolated from the four closest weather stations, adjusted for elevation and location differentials with regional gradients and using a 1/*d* ^2^ weighted average, where *d* is the distance between the provenance and station locations. Climate data were obtained by using the network of Environment Canada weather stations (Environment Canada, 2013), as well as the Ministère de l'Environnement et de la Lutte contre les Changements Climatiques du Québec, the Centre informatique de prévision des ravageurs en agriculture, and the Solution‐Mesonet station networks (Lepage & Bourgeois, 2011). Interpolated climate data for the common garden site covered the period 1989–2007, and data for the 43 provenance origins covered the 1950–1980 period, which broadly corresponds to the climate of the 25 yr period preceding seed collection in the natural populations. Annual and/or monthly values of climate variables were defined following the Climdex database (http://www.climdex.org/indices.html). The description of the estimated climate variables and their abbreviations are reported in Table 1. In addition, the soil moisture index (SMI, available soil moisture) was estimated for each month using the quadratic + linear formulation procedure, which accounts for water loss through evapotranspiration (simplified Penman–Monteith potential evapotranspiration) and water gain from precipitation (Hogg *et al.*, 2013). Parameter values for maximum and critical available soil water were set at 300 mm and 400 mm, respectively. One should note that SMI is based on climatic physics alone, and thus not fully representative of actual *in situ* soil moisture conditions. Climate regime data for the Mastigouche common garden site and the 43 geographic origins of the provenances are summarized in Table S1.

**Table 1:** List of abbreviations and definitions of tree‐ring traits and climatic variables presented in this study.

Variable name	Unit	Description
**Tree‐ring traits**		
*Wood traits*		
RW	mm	Ring width
BAI	mm^2^	Basal area increment (growth performance in this study)
WD	kg·m^−3^	Wood density
CWT	µm	Cell wall thickness
LD	µm	Average lumen diameter
CWR	unitless	Average conduit wall reinforcement
LDr	µm	Radial lumen diameter
CWRr	unitless	Radial conduit wall reinforcement
*Drought‐resilience traits*		
Rs	unitless	Resistance of a tree in response to a periodic drought
Rc	unitless	Recovery of a tree in response to a periodic drought
Rl	unitless	Resilience of a tree in response to a periodic drought
Rr	unitless	Relative resilience of a tree in response to a periodic drought
*Climate sensitivity traits*		
COR*_X_*_–_ *_Y_*_(_ *_t_*_)_	unitless	Climate sensitivity traits used in this study. Coefficient of correlation between a wood trait *X* and a climatic variable in month *Y* (month of the previous year and the contemporaneous year being labelled (*t* − 1) and (*t*), respectively)
**Climatic variables**		
ADD	days	Number of days in the year when the daily precipitation is < 0.2 mm
MAT	°C	Mean annual temperature
MAP	mm	Mean annual precipitation
SMI	%	Soil moisture index
Summer_SMI	%	Summer mean (June, July, August) of the soil moisture index
*T*_min_	°C	Minimum monthly‐based temperature
*T*_max_	°C	Maximum monthly‐based temperature

### Characterization of wood traits in tree rings

The definitions and abbreviations of wood traits presented in this study are detailed in Table 1. A 12 mm wood increment core was extracted around breast height (120 cm from the ground) from each tree in 2006, 27 yr after the establishment of the common garden study, and was then processed as described in Beaulieu *et al. *(2014). Wood trait data were obtained by combining image analysis with X‐ray densitometry and diffractometry at the FPInnovations facilities (Vancouver, BC, Canada) using the SilviScan technology. The ring width (RW), radial and tangential tracheid diameters, and cell‐wall thickness (WT; Fig. S2) were measured from the pith to the bark along the southern radius of each core for consecutive radial intervals of 25 μm. WD was measured by X‐ray densitometry at a sampling interval of 25 μm as previously described (Beaulieu *et al.*, 2014). Cross‐dating of 1481 individual tree‐ring series was verified using cofecha (Holmes, 1983; Grissino‐Mayer, 2001).

RW was transformed into basal area increment (BAI; bai.out function of the dplr package, R v.3.3.4, R Core Team, 2014; Bunn, 2008). Proxies reflecting hydraulic performance such as lumen diameters (LDs) and cell‐wall reinforcement (CWR) were derived from the obtained wood traits. Briefly, radial and tangential LDs (LDr and LDt) were estimated as LDr = TDr − CWT and LDt = TDt − CWT, where TD is the tracheid diameter and CWT is the cell‐wall thickness (for details see Fig. S2). The CWR, a surrogate of xylem resistance to drought‐induced embolism, was also calculated based on LDs and CWT (Fig. S2; Hacke *et al.*, 2001; Rosner *et al.*, 2016). Finally, average LD and CWR were calculated as the mean value of the radial and tangential parameters. Basic statistics for wood trait comparisons among populations are reported in Table S2.

### Tree growth response following periodic drought events

**Fig. 2:**
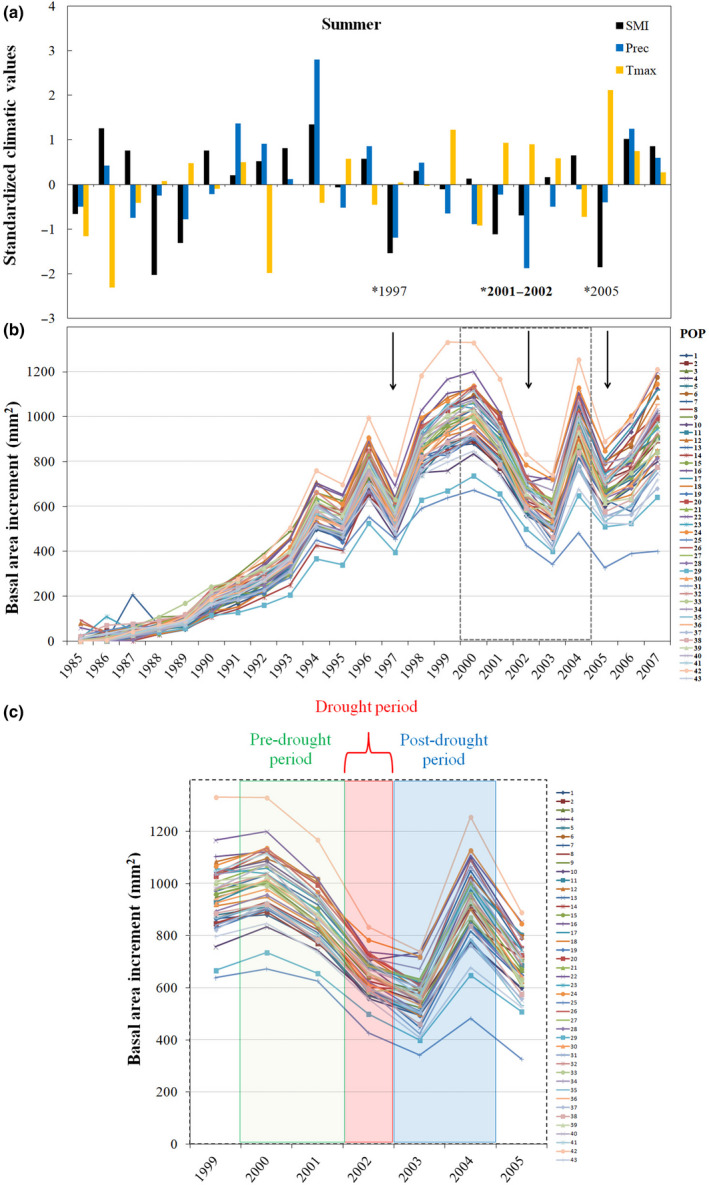
Climate variation and basal area increments (BAIs) of white spruce provenances. (a) Temporal variation of mean summer (June, July and August) maximum temperatures *T*_max_, total precipitation (Prec) and soil moisture index (SMI) from 1985 to 2007 at the common garden site. Climate deviations from the long‐term mean were estimated as the ratio between the annual mean summer value and the mean summer value for the period 1989–2007. (b) Annual variation in radial tree growth increment (BAI) for the period 1985–2007. Arrows indicate drought years that coincide with abrupt decreases in BAI. (c) Annual variation in radial tree growth increment (BAI) for the period 1999–2005. The different time periods used to calculate the drought‐resilience traits in 2002 are indicated by different colours.

Climate deviations from the long‐term mean for the period studied (1989–2007) revealed the presence of three extreme drought episodes (1997, 2001–2002 and 2005; see Figs 2a, S3) that paralleled abrupt reductions in annual growth (Fig. 2b). For each drought event, four different indices, resistance (Rs), recovery (Rc), resilience (Rl), and relative resilience (Rr), were calculated from BAI values using the pointres R package (van der Maaten‐Theunissen *et al.*, 2015). Lloret *et al. *(2011) introduced the concepts of tree drought‐resistance (Rs; the ability of a tree to resist growth reduction under drought) and tree recovery (Rc; the ability of a tree to recover its growth after stress) (Fig. 1). Resilience (Rl) is generally defined as the ability of a tree to return to pregrowth rates following a drought event. Resistance (Rs) was estimated as Dr/PreDr, where Dr and PreDr are BAI during and before the drought event, respectively. Recovery (Rc) is calculated as PostDr/Dr, where PostDr is the BAI following the event. Resilience (Rl) was calculated by dividing post‐drought BAI by pre‐drought BAI. The relative resilience (Rr) index takes into account the severity of the abrupt growth reduction and was determined by subtracting Rs from Rl. The drought event was defined as occurring over a 1 yr period (in 1997, 2002 and 2005; see Fig. 2b,c), whereas pre‐drought and post‐drought events each covered a 2 yr period. The four resilience indices (Rs, Rl, Rc and Rr) were averaged over the three drought events to estimate long‐term resilience (referred to as Rs_MEAN_, Rl_MEAN_, Rc_MEAN_ and Rr_MEAN_). Given the less intense nature of the 1997 and 2005 drought episodes compared with 2001–2002, we will only report growth responses for the spatially extensive and long‐lasting 2001–2002 drought, as well as the average growth responses over the three drought events (Wheaton *et al.*, 2008). The drought indices reflecting the short‐term and long‐term resilience capacity of white spruce trees (Rs, Rl, Rc, Rr, and their averages) are referred to as drought‐resilience (DR) traits.

Linear mixed models were used to examine the effects of provenance, family and tree size on the DR traits. Models were fitted using the ‘asreml’ function from the asreml‐r v.4.0 R package (Butler *et al.*, 2017) as follows:

(Eqn 1) $$Y_{\mathrm{ijkl}}=\mu +{\mathrm{block}}_{k}+{tree_{s}ize}_{l}+{\mathrm{pop}}_{i}+\mathrm{fam}{\left(\mathrm{pop}\right)}_{j(i)}+{\mathrm{fam}}_{j}:{\mathrm{block}}_{k}+e_{\mathrm{ijkl}}$$

where *Y_ijkl_* is the drought index value on the *l* ^th^ tree from the *j* ^th^ family within the *i* ^th^ population (i.e. provenance) recorded in the *k* ^th^ block of the common garden; *µ* is the overall mean. The block and tree size effects, block*_k_* and tree_size*_l_*, were considered as fixed effects in the model. The covariable tree_size*_l_* refers to the sum of basal area increment (in 2002 for DR traits estimated in 2002, or at the time of harvest for the long‐term DR traits). pop*_i_* and fam(pop)*_j_*_(_ *_i_*_)_ are the random effects of population and family within population, respectively; fam*_j_* : block*_k_* refers to the random effect of family‐by‐block interaction, which is the plot error term; *e_ijkl_* is the error term.

### Relationships between wood traits and climate in the common garden site

Dendroclimatic analyses were conducted to determine which climate variables measured at the common garden site were most strongly associated with tree growth patterns and the studied wood traits (i.e. BAI, CWT, WD, LD_r_ and CWR_r_). Wood trait time series were standardized in a two‐step procedure (Methods S1). First, the effects of nonclimate factors (e.g. age‐related trend, size‐related trend and competition effect) were removed using Generalized Additive Mixed Models (GAMMs; *bam* function in the mgcv R package; Wood, 2011). For this approach, each phenotypic trait was modelled as a function of cambial age, tree basal area, and competition. Second, autocorrelations present within annual changes in wood traits of individual cores were removed using the autoregressive ‘AR model’ function (detrender package; Bunn, 2008). General statistics for residuals were then computed using the detrender package. The expressed population signals (EPS) of all studied traits were higher than 0.85. The residuals from the trait time series were averaged to construct mean residual chronologies for each provenance. We excluded residual growth data before 1989 to avoid the more juvenile growth stage during the first ten years of growth in the common garden when age/size strongly influences spruce growth (Fig. 2b). Dendroclimatic relationships between residual chronologies of wood traits and monthly values of climate variables at the common garden site for the period 1989–2007 were then examined (treeclim R package; Zang & Biondi, 2015). The climate‐sensitivity traits (i.e. COR*_X_*_–_ *_Y_*_(_ *_t_*_)_, see Table 1) were defined as the Pearson correlation coefficients between the mean residual chronologies (means per population per year) and a given climate variable (Fig. S2). Climate variables from May of the previous year until October of the current year were considered as potentially affecting tree growth and wood traits for a given year (Huang *et al.*, 2010).

### Relationships between tree‐ring traits and climate at provenance origin

Multivariate adaptive regression splines (MARS; Milborrow, 2011) were fitted to explore the relationship between tree‐ring traits (i.e. wood traits, DR traits and climate sensitivity traits) and the climate of the 43 provenance origins for the period 1950–1980. MARS is a nonparametric regression procedure that does not make assumptions about the underlying functional relationships between the dependent and independent variables (Friedman, 1991). MARS is a form of piecewise regression where the relationships between response and explanatory variables are described by a series of linear segments of differing slopes, each of which was fitted using a basis function. Breaks between segments were defined by a knot in the model that initially overfitted the data and was then simplified using a backward/forward stepwise cross‐validation procedure. The long‐term growth performance of individual trees was calculated based on average annual radial growth rates (BAI) for the 1995–2005 period. The following climate variables calculated over the period 1950 to 1980 for each of the 43 provenance origins were identified as potentially biologically relevant for population differentiation and genetic adaptation to local conditions: mean annual temperature (MAT), mean annual precipitation (MAP), annual count of dry days (ADD), and soil moisture index in summer (Summer_SMI). The R package earth was used for MARS modelling (R Development Core Team, 2014).

### Heritability and among‐population genetic differentiation

For each trait, heritability and among‐population genetic differentiation estimates were derived from a linear mixed model using the R package asreml‐r v.4.0 (Butler *et al.*, 2017):

(Eqn 2) $$Y_{\mathrm{ijklm}}=\mu +{\text{year}}_{m}+{\text{block}}_{k}\left({\text{year}}_{m}\right)+{\text{pop}}_{i}+\text{fam}{\left(\text{pop}\right)}_{j(i)}+{\text{pop}}_{i}:{\text{year}}_{m}+\text{fam}{\left(\text{pop}\right)}_{j(i)}:{\text{year}}_{m}+{\text{fam}}_{j}:{\text{block}}_{k}\left({\text{year}}_{m}\right)+e_{\mathrm{ijlkm}}$$

where*Y_ijklm_* is the observation on the *l* ^th^ tree in the *m* ^th^ year from the *j* ^th^ family within the *i* ^th^ population (i.e. provenance) recorded in the *k* ^th^ block of the common garden; *µ* is the overall mean; yea*r_m_* is the fixed time effect; block*_k_*(year*_m_*) is the fixed effect of the block nested within year; pop*_i_* is the random population effect with ${\text{pop}}_{i}∼N\left(0,\sigma_{\text{pop}}^{2}\right)$; fam(pop)*_j_*_(_ *_i_*_)_ is the random family effect nested within population, with $\text{fam}{\left(\text{pop}\right)}_{j(i)}∼N\left(0,\sigma_{\text{fam}(\text{pop})}^{2}\right)$; pop*_i_* : year*_m_* is the random population by year interaction, with ${\text{pop}}_{i}:{\text{year}}_{m}∼N\left(0,\sigma_{\text{pop}:\text{year}}^{2}\right)$; fam(pop)*_j_*_(_ *_i_*_)_ : year*_m_* is the random family within population by year interaction, with $\text{fam}{\left(\text{pop}\right)}_{j\left(i\right)}:{\text{year}}_{m}∼N\left(0,\sigma_{\text{fam(pop)}:\text{year}}^{2}\right)$; and fam*_j_* : block*_k_*(year*_m_*) is the random family by block interaction (i.e. the plot effect) nested within year, with ${\text{fam}}_{j}:{\text{block}}_{k}\left({\text{year}}_{m}\right)∼N\left(0,P\right)$, where ***P*** is a block diagonal matrix with a different (‍ $\sigma_{\text{fam}:\text{block(year)}}^{2}$‍) variance for each year. The year*_m_* effect was considered as a fixed effect in our models. The block and plot effects (i.e. ${\text{fam}}_{j}:{\text{block}}_{k}$‍) were nested within year to incorporate the different effects at each time point (Gezan & Carvalho, 2018). Given the repeated measures on the same increment core, the error term was assumed to be $e∼N(0,I_{l}⊗R_{m})$, where ***I*** *_l_* is an identity matrix of dimension *l* (1481 trees), ***R*** *_m_* represents a first‐order heterogeneous autoregressive correlation structure (ARH1) on time of dimension *m* × *m* (11 yr × 11 yr), and the symbol ⊗ refers to the Kronecker product. Because the DR traits were analysed for distinct years, the year*_m_* effect and its interaction terms were omitted from the model, and the error term was specified as $e_{\mathrm{ijklm}}∼N\left(0,\sigma_{e}^{2}\right)$. When necessary, data were transformed to stabilize the variances (Table 2). Model parameters were estimated with the restricted maximum likelihood method (REML).

**Table 2:** Variance components for the wood and drought resilience traits studied within and among white spruce provenances.

Trait^1^	${\hat{\sigma }}_{\text{pop}}^{2}$	${\hat{\sigma }}_{\text{fam(pop)}}^{2}$	${\hat{\sigma }}_{p}^{2}$	$h_{\text{ind}}^{2}$ (95% CI)	*Q*_ST_ (95% CI)	Data transformation
BAI	0.008	0.005	0.302	0.067 (−0.024, 0.158)	0.164 (−0.077, 0.406)	log
WD	110.689	144.723	1689.941	0.333 (0.217, 0.448)	0.090 (0.026, 0.154)	—
CWT	0.010	0.015	0.179	0.335 (0.215, 0.454)	0.078 (0.018, 0.139)	—
LDr	0.283 × 10^−3^	0.796 × 10^−3^	0.007	0.412 (0.267, 0.557)	0.044 (−0.002, 0.089)	log
LD	0.123	0.204	2.95	0.269 (0.143, 0.394)	0.072 (0.004, 0.140)	—
CWRr	0.004	0.008	0.079	0.416 (0.286, 0.545)	0.064 (0.013, 0.114)	log
CWR	0.004	0.005	0.062	0.341 (0.223, 0.460)	0.090 (0.025, 0.154)	log
Rs_2002_	1.363 × 10^−9^	4.677 × 10^−4^	0.052	0.112 (−0.025, 0.249)	3.830 × 10^−7^ (−9.131 × 10^−8^, 0.573 × 10^−7^)	—
Rl_2002_	0.161 × 10^−2^	0.347 × 10^−2^	0.045	0.301 (0.099, 0.496)	0.056 (−0.018, 0.132)	—
Rc_2002_	0.303 × 10^−2^	0.570 × 10^−2^	0.066	0.337 (0.149, 0.524)	0.064 (−0.008, 0.136)	—
Rr_2002_	0.169 × 10^−2^	0.287 × 10^−2^	0.032	0.352 (0.097, 0.541)	0.071 (−0.004, 0.145)	—

Variance among provenances ${\hat{\sigma }}_{\text{pop}}^{2}$‍, family within provenance genetic variance ${\hat{\sigma }}_{\text{fam(pop)}}^{2}$‍, and the phenotypic variance ${\hat{\sigma }}_{p}^{2}$ (with ${\hat{\sigma }}_{p}^{2}={\hat{\sigma }}_{\text{fam}:\text{block(year)}}^{2}+{\hat{\sigma }}_{\text{fam(pop)}}^{2}+\sigma_{\text{fam(pop)}:\text{year}}^{2}+{\hat{\sigma }}_{e}^{2}$‍) are reported. For each tree‐ring trait, the index of phenotypic differentiation *Q*_ST_ and the narrow‐sense heritability $h_{\text{ind}}^{2}$ are reported. Values in parentheses represent the 95% confidence intervals (see the Materials and Methods section). When applicable, the type of data transformation is reported in the last column.

^1^ BAI, basal area increment; WD, wood density; CWT, cell wall thickness; LDr, radial lumen diameter; LD, average lumen diameter; CWR, cell wall reinforcement or wood density; CWRr, radial cell‐wall reinforcement; Rs_2002_, growth resistance; Rl_2002_, growth resilience; Rc_2002_, growth recovery; Rr_2002_, growth relative resilience.

After extracting the variance components of the above‐mentioned model, the index of among‐population differentiation *Q*_ST_ was estimated for each trait according to:

(Eqn 3) $$Q_{\text{ST}}={\hat{\sigma }}_{\text{pop}}^{2}/\left({\hat{\sigma }}_{\text{pop}}^{2}+\frac{2}{r}\right){\hat{\sigma }}_{\text{fam}(\text{pop})}^{2}$$

where *r* is the average coefficient of relatedness between trees. The individual narrow‐sense heritability was estimated as:

(Eqn 4) $$h_{\text{ind}}^{2}=\frac{1}{r}{\hat{\sigma }}_{\text{fam(pop)}}^{2}/\left({\hat{\sigma }}_{\text{fam(pop)}}^{2}+\sigma_{\text{fam(pop)}:\text{year}}^{2}+{\hat{\sigma }}_{\text{fam}:\text{block(year)}}^{2}+{\hat{\sigma }}_{e}^{2}\right)$$

For this calculation, ${\hat{\sigma }}_{\text{fam}:\text{block(year)}}^{2}$ and ${\hat{\sigma }}_{e}^{2}$ were averaged across years. The SEs of $h_{\text{ind}}^{2}$ and *Q*_ST_ estimates were obtained using the delta method, and confidence intervals were deduced from SEs (1.96 × SE). When the progeny of each maternal tree are all half‐siblings, the average coefficient of relatedness is assumed to be *r* = 0.25. However, since paternity is unknown in our data set, some of the families may contain full siblings (*r* = 0.5). Thus, by setting *r* to 0.25, the additive genetic variance and heritability are likely overestimated and *Q*_ST_ values are underestimated. To avoid such bias, we estimated the average genomic relationship between trees within families in our data set. To do so, the realized genomic relationship matrix (***G***‐matrix) was computed from 6386 validated single nucleotide polymorphism (SNP) markers (Pavy *et al.*, 2013) using the ‘A.mat’ function of the rrblup R package (Endelman and Jannink, 2012) with the default options. The estimated average genomic relationship between trees within families (*r* = 0.2573) was further used in Eqns 3 and 4.

For each of the 6386 SNP markers, basic statistics including the population differentiation due to genetic structure *F*_ST_ (Table S3) were obtained using the adegenet and hierfstat R packages (Goudet, 2005; Jombart, 2008). The estimated neutral genetic divergence among provenances *F*_ST_ was 0.0430.

### Data availability

The original phenotypic data are part of the network of the Natural Resources Canada white spruce genecological tests and have been stored in our institution’s database (https://treesource.rncan.gc.ca). Full access can be shared upon request to the corresponding author according to the intellectual property policies of participating governmental institutions. The data and R scripts are available on the Github website (https://github.com/ClaireDepardieu/Resilience_white_spruce). Genotyping data are accessible through the Dryad Digital Repository (https://doi.org/10.5061/dryad.6rd6f).

## Results

### Growth performance and relationships with climate at provenance origin

Significant differences in BAI were observed among populations at the common garden site (*P* < 0.001; Table S2). POP_25 was identified as the least productive, and POP_42 performed best (Fig. S4). Relationships between population means of BAI and climate at provenance origin for the period 1950–1980 revealed that MAT, soil moisture in summer (Summer_SMI), and the annual dry days (ADD) were significant predictors of BAI, with white spruce populations from drier climates showing greater growth rates (Fig. S5). Maximum BAI occurred in populations originating from cooler climate locations (MAT *c.* 1°C cooler) compared with the common garden site (Fig. S5a), while maximum radial growth occurred in populations from drier climate locations than the common garden site (Fig. S5b,c).

### Relationships between wood traits and climate at the common garden site

**Fig. 3:**
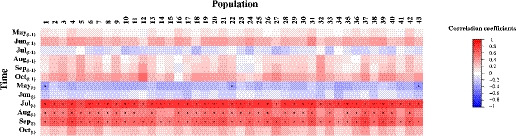
Climate–growth associations of white spruce provenances. Correlation analyses of basal area increment residual chronologies against the monthly mean soil moisture index (SMI) from May to October of the previous (*t* − 1) growing season and the current growing season (*t*) are presented for the period 1989–2007. The scale bar reports positive (red) and negative (blue) correlation coefficients. Significant relationships (*P < *0.05) are indicated by dots in the correlation matrix.

Dendroclimatic analyses revealed that wood traits were affected by soil moisture (Fig. 3), which was influenced by precipitation and temperature (Fig. S3). Growth–climate correlations across tree lifespan (i.e. climate‐sensitivity traits) highlighted a positive and significant relationship between BAI and SMI during the current growing season (i.e. in July_(_ *_t_*_)_, August_(_ *_t_*_)_ and September_(_ *_t_*_)_; Fig. 3). Decreases in radial growth under drought conditions were explained by the formation of tracheids with smaller LDs in July (LDr; Fig. S6a), resulting in a significant increase in CWR_r_ and WD for several populations (Fig. S6c,d). The climate data revealed the occurrence of three major drought events (1997, 2001–2002, 2005) during the period studied (Figs 2a, S3). Drought conditions likely originated from a combination of high maximum temperatures and a lack of precipitation in July_(_ *_t_*_)_, both having a negative impact on growth and cell morphology (Figs S3, S6).

With two consecutive years of severe water deficits recorded in 2001–2002, this drought event was considered the most extreme (2001: August and September; 2002: September and October; Fig. S3c). For the 2001–2002 drought event, the provenance effect was highly significant for the DR traits, recovery (Rc), resilience (Rl) and relative resilience (Rr) while a smaller but significant provenance effect was found for resistance (Rs; Table S4). In particular, POP_25 (a southern provenance) exhibited the lowest recovery rates and POP_10 (a provenance originating from a dry location) had the highest recovery rates after 2002 (Fig. S7a). The most resilient populations exhibited higher recovery rates following drought and were the most productive at the common garden site (Fig. S8). Significant family effects were also detected for all the DR traits for the 2001–2002 drought event (Table S4).

### Clinal variation along soil water availability and temperature gradients

**Fig. 4:**
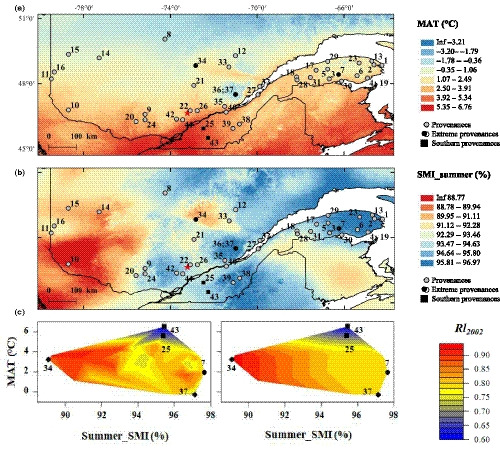
Growth resilience in relation to climate at white spruce provenance origin. Maps of growth resilience (Rl) against (a) the mean annual temperature (MAT) and (b) mean summer soil moisture index (Summer_SMI) for the period 1950–1980. (c) Multivariate adaptive regression spline (MARS) results presenting growth resilience (Rl_2002_) as a function of both MAT and Summer_SMI. A gridded bivariate spline interpolation was applied to the irregularly spaced observed (left) and predicted (right) resilience data. The extreme provenances along the aridity gradient (i.e. POP_34, POP_37 and POP_7) identified for the observed and predicted Rl_2002_ values are represented by black circles. The two southern provenances (i.e. POP_25 and POP_43) are represented by black squares. The scale bar indicates low (blue), medium (yellow) and high (red) correlation coefficients. MARS analyses revealed that both Summer_SMI and MAT were significant predictors of growth resilience. Rl_2002_ was positively correlated to soil moisture in summer (provenances shown in circles), whereas the lowest resilience for POP_43 and POP_25 (provenances shown in black squares) was mainly explained by temperature.

Clinal variation was tested between the DR traits (i.e. Rl_2002_, Rc_2002_ and Rr_2002_) and local climate conditions at provenance origin. MARS (a form of regression analysis that considers nonlinearities and interactions between climate variables) showed that Summer_SMI and MAT at provenance origin were significant climate predictors for all traits tested (Fig. 4; Notes S1). In particular, the combination of MAT and Summer_SMI explained 57.1% of the variation observed in Rl_2002_ (Fig. 4c; Notes S1). For values of MAT > 5°C, values of Rl_2002_ increase rapidly with decreasing Summer_SMI, whereas the increase of Rl_2002_ with decreasing Summer_SMI is less for low MAT values (Fig. 4c). Low Rl_2002_ values for POP_25 and POP_43 were mainly explained by high MAT at provenance origin, whereas provenances from drier environments were typically associated with higher growth resilience values.

### Genetic differentiation among populations and genetic control of wood traits

For most of the traits tested, a significant proportion of variance was associated with differences among families ( ${\hat{\sigma }}_{\text{fam(pop)}}^{2}$‍; Table 2)_._ Narrow‐sense heritability estimates $h_{\text{ind}}^{2}$ indicated that quantitative traits were under low to moderate genetic control (‍ $0.067\leq h_{\text{ind}}^{2}\leq 0.412$‍), with LDr and CWRr showing the highest values. Notably, all drought‐resilience traits (i.e. Rs_2002,_ Rl_2002_, Rc_2002_ and Rr_2002_) exhibited higher $h_{\text{ind}}^{2}$ estimates (‍ $0.112\leq h_{\text{ind}}^{2}\leq 0.352$‍) compared with radial growth rate (BAI). The index of among‐population differentiation in quantitative traits *Q*_ST_ ranged from 3.83 × 10^−7^ to 0.090 and revealed significant genetic variation among populations for WD, CWT, LD, CWRr and CWR (Table 2). In particular, WD and CWR had the highest differentiation, with *Q*_ST_ ≥ 0.08.

## Discussion

### A local genetic drought‐adaptation signal detected in tree responses

We found that soil moisture availability explains a significant portion of growth variation in white spruce (Figs 2, 3), which is in line with previous studies (Barber *et al.*, 2000; Chen *et al.*, 2017). Interestingly, we showed that genetics plays a major role in determining growth responses to drought at the intraspecific level (Table 2). In this study, which considered a widely distributed northern conifer, we evidenced that adaptive genetic variation in response to geographically variable climatic conditions can occur, likely reflecting local adaptation and an increased resilience to drought (Fig. 4c). In our tree‐ring series, the consistent radial growth decrease observed in 2002 may be explained by the additional effect of major droughts in 2001 and 2002 (Fig. 2a). Since carbohydrates and water reserves are generally mobilized during bud break to sustain spring growth, a severe late‐season drought that reduces reserves and affects partitioning can influence growth rates the following spring (Adams *et al.*, 2009; Babst *et al.*, 2012). The analysis of growth responses in relation to climate at provenance origin revealed clinal trends in population variation, with populations from locations with drier climates showing greater resilience than those from more humid locations (Fig. 4). A similar genetic adaptation trend has been reported at the interspecific level, where coniferous species from dry regions in southwestern USA and southern Europe exhibited better growth recovery than those from wetter regions (Gazol *et al.*, 2016; Sánchez‐Salguero *et al.*, 2018). Except for Rs_2002_, all DR traits were positively correlated with mean BAI, revealing their potential in predicting the long‐term productivity of relatively young white spruce trees (Fig. S8; Table S5). This result was unexpected, given that no differences among white spruce populations had been previously detected for other anatomic hydraulic‐related traits associated with drought tolerance (Sebastian‐Azcona *et al.*, 2018). Several studies have shown that trees with slower growth rates are more susceptible to drought, implying that mean growth rate is a good predictor of survival (Suarez *et al.*, 2004; Lloret *et al.*, 2011). In addition, the generally higher recovery capacity of trees from the most productive provenances in our study, those from drier climates (Fig. 4), indicates that these provenances are likely to better acclimate and cope with drought. Growth response to drought was also significantly affected by the mean annual temperature at provenance origin, with the two southern populations POP_25 and POP_43 exhibiting the lowest resilience.

In our study, provenances originating from drier locations had lower WD, larger tracheid lumens, and thinner cell walls (Table S5). This adaptation in xylem structure may be interpreted as a means of increasing hydraulic efficiency with minimal C costs in areas where photosynthetic rates may be limited due to frequent drought events. Our results confirm previous observations made on pines (Martín *et al.*, 2010) and are congruent with the finding that efficient water transport is associated with vulnerable xylem cavitation and low WD in white spruce trees, which indicates the existence of a trade‐off between water transport efficiency and resistance to cavitation (Sebastian‐Azcona *et al.*, 2018).

Different morphological and physiological processes may explain the differences we observed in post‐drought growth recovery among provenances. It is generally understood that the ability of trees to survive and recover from drought relies on the balance between C gain and water loss (McDowell, 2011). In our study, different root architectures and/or water use efficiencies among provenances may have affected their ability to maintain water uptake and transport during drought episodes. Several studies showed that post‐drought growth recovery can be related to stem C reserves, an optimal C allocation allowing trees to regrow drought‐damaged xylem and repair water transport tissues (Galiano *et al.*, 2011; Trugman *et al.*, 2018). More generally, since information is scarce regarding various hydraulic strategies related to DR in conifers, further research is needed to determine how contrasting white spruce provenances and families prioritize C gain and hydraulic vulnerability under water‐deficit conditions in relation to their differing abilities to recover growth following severe drought conditions.

### Building growth resilience under climate change conditions: possibilities and challenges

The possibility of lowering drought sensitivity in coniferous plantations and ecosystems is crucial for maintaining ecosystem health, productivity, and services under predicted environmental changes. The selection of less sensitive seed sources for reforestation or the consideration of resistance traits in breeding objectives represent approaches now available to forest managers. Both climate sensitivity and genetic variability of hydraulic‐related wood traits are important aspects to consider when studying drought adaptation in tree species (Rosner *et al.*, 2014; George *et al.*, 2017). In line with recent observations at the intraspecific level and ecosystem scale (George *et al.*, 2015; Anderegg *et al.*, 2018), WD in our study was a poor predictor of DR (i.e. short‐term and long‐term DR traits versus WD; Table S5). Instead, the consistent relationships found between mean growth recovery (Rc_MEAN_) and drought sensitivity of CWR over the period studied (‍ ${\text{COR}}_{{\text{CWR}}_{r}{\text{-July}}_{\left(t\right)}}$_‍_; Table S5) suggest that white spruce provenances are able to modulate their xylem anatomy under water stress, which may have an impact on DR. The traits examined in this study were under moderate genetic control (Table 2), suggesting that breeding and selection could be pursued to improve these traits. Interestingly, our data set does not reveal any trade‐offs between increased DR and growth (Fig. S8). Breeding for improved DR traits would thus not jeopardize wood production in future plantations. Hence, DR traits may represent a new suite of traits that could be used to improve the resilience of future plantations in the face of projected frequent and hotter droughts in northeastern North America. The moderate heritability estimates for Rc_2002,_ Rl_2002_ and Rr_2002_ and the noted genetic differentiation among provenances lead us to expect significant genetic gains from including these traits in selection schemes. In addition, our results reveal that high genetic variation for drought resistance exists among families within provenances ( ${\hat{\sigma }}_{\text{fam(pop)}}^{2}$ vs ${\hat{\sigma }}_{\text{pop}}^{2}$‍; Table 2), suggesting a multistage selection strategy to capture the various sources of genetic variation identified.

Since our data set did not include provenances spanning the entire natural distribution of white spruce, additional studies should be initiated in different ecological regions using provenances spanning the entire environmental range of the species in order to identify resilient provenances and families and to monitor rank changes in selections across different environments. In particular, the range of white spruce extends well into the vast Canadian Prairies, where the climate is typically drier than in eastern Canada and where steeper clines may be detected in relation to DR traits. Such an endeavour represents a daunting task, given the relative lack of adequate provenance tests in many regions and the great efforts needed to establish new common garden field trials with provenances from various regions and monitoring of tree growth over many years.

### Tree rings or biological archives: understanding the past to better predict the future

One major outcome from this work was the surprising impact of episodic climate disturbances – presumably as important as incremental changes in climatic conditions – on tree survival and fitness. These findings reconcile well with the notion that infrequent but extreme events can be highly consequential to natural selection in boreal conifers, especially given their long development period before attaining sexual maturity. Although they are much less diversified than angiosperms and bear more archaic features, conifers have existed for more than 250 Myr, spanning periods warmer than the present (Gernandt *et al.*, 2011). They have been shown to evolve simple but efficient mechanisms to resist drought, thereby likely contributing to their enduring existence on the planet (Brodribb *et al.*, 2010). Our findings support the idea that, within certain limits, conifers are genetically programmed for this drought response, thereby indicating that adaptive genetic variation also exists within species in response to variable local conditions. Such intraspecific variation should endow these species with a greater ability for survival and long‐term persistence. Ultimately, this study also illustrates how dendroecology and quantitative genetics can be cross‐utilized to deepen our understanding of the genetic basis of drought adaptation throughout tree lifespans, as well as how long‐lived species can survive and adapt to the increasing instability of local climates. Recent studies in conifers underlined the potential of combining dendroclimatic, phenotypic and genomic data to gain insight into the genetic basis of intraspecific variation in drought sensitivity and local adaptation to climate change (Housset *et al.*, 2018; Trujillo‐Moya *et al.*, 2018). In the context of a progressively more drought‐prone environment, such an integrated research approach is expected to improve conventional tree breeding, along with other approaches such as genomic selection (Park *et al.*, 2016; Grattapaglia *et al.*, 2018) and high‐throughput phenotyping (D’Odorico *et al.* 2020), with the aim of developing mitigating solutions and sustaining productivity in forest tree species.

## Author contributions

NI, CD, PL and MPG designed the study and methodology. CD, MPG, SN, JB and PL performed the analyses and discussed the results. CD wrote the manuscript draft with inputs from NI, JB, MPG, SN and PL.

## Supporting information

**Fig. S1** Geographic location of the 43 provenances sampled.
**Fig. S2** Schematic representation of the tree‐ring traits examined in this study.
**Fig. S3** Monthly variation of total precipitation (MAP), maximum temperature (Tmax) and soil moisture index (SMI) for the 1996–2008 period.
**Fig. S4** Growth performance of white spruce seed provenances at the common garden site.
**Fig. S5** Provenance means for basal area increment (BAI) plotted against mean annual temperature (MAT), summer soil moisture index (Summer_SMI), and annual number of dry days (ADD) at provenance origin.
**Fig. S6** Impact of drought on cell morphology.
**Fig. S7** Box plots for growth recovery and growth resilience for the 2001–2002 drought event.
**Fig. S8** Relationship between radial growth (mean BAI for each provenance) and growth recovery, growth relative resilience, and growth resilience.
**Methods S1** Calculation of the competition index and detrending methods.
**Notes S1** Relationship between the drought‐resilience traits (i.e. Rc2002, Rl2002 and Rr2002) and the climatic variables at provenance origins.
**Table S1** Mean annual bioclimatic characteristics of the 43 provenances and the common garden site over the 1950–1980 period.
**Table S2** Basic statistics for the studied wood traits.
**Table S3** Basic statistics estimated per SNP for the 6386 SNPs used in the present study. See separate file.
**Table S4** Linear modeling analysis for long‐term and the 2002 drought‐resilience (DR) traits.
**Table S5** Pairwise Pearson correlations between the studied traits.
Please note: Wiley Blackwell are not responsible for the content or functionality of any Supporting Information supplied by the authors. Any queries (other than missing material) should be directed to the *New Phytologist* Central Office.
