# Supplementary material for: Adaptive genetic variation to drought in a widely distributed conifer suggests a potential for increasing forest resilience in a drying climate
Source: New Phytol. 2020 May 12;227(2):427–39. doi: 10.1111/nph.16551 (PMC7317761; doi:10.1111/nph.16551)
Supplement: Supplementary file 2 — Fig. S1 Geographic location of the 43 provenances sampled. Fig. S2 Schematic representation of the tree‐ring traits examined in this study. Fig. S3 Monthly variation of total precipitation (MAP), maximum temperature (Tmax) and soil moisture index (SMI) for the 1996–2008 period. Fig. S4 Growth performance of white spruce seed provenances at the common garden site. Fig. S5 Provenance means for basal area increment (BAI) plotted against mean annual temperature (MAT), summer soil moisture index (Summer_SMI), and annual number of dry days (ADD) at provenance origin. Fig. S6 Impact of drought on cell morphology. Fig. S7 Box plots for growth recovery and growth resilience for the 2001–2002 drought event. Fig. S8 Relationship between radial growth (mean BAI for each provenance) and growth recovery, growth relative resilience, and growth resilience. Methods S1 Calculation of the competition index and detrending methods. Notes S1 Relationship between the drought‐resilience traits (i.e. Rc2002, Rl2002 and Rr2002) and the climatic variables at provenance origins. Table S1 Mean annual bioclimatic characteristics of the 43 provenances and the common garden site over the 1950–1980 period. Table S2 Basic statistics for the studied wood traits. Table S3 Basic statistics estimated per SNP for the 6386 SNPs used in the present study. See separate file. Table S4 Linear modeling analysis for long‐term and the 2002 drought‐resilience (DR) traits. Table S5 Pairwise Pearson correlations between the studied traits. Please note: Wiley Blackwell are not responsible for the content or functionality of any Supporting Information supplied by the authors. Any queries (other than missing material) should be directed to the New Phytologist Central Office. [file NPH-227-427-s002.pdf]

New Phytologist

***New Phytologist* Supporting Information**

Article title: **Adaptive genetic variation to drought in a widely distributed conifer suggests a potential for increasing forest resilience in a drying climate**

Authors: Claire Depardieu, Martin P. Girardin, Simon Nadeau, Patrick Lenz, Jean Bousquet, Nathalie Isabel

Article acceptance date: 29 February 2020

The following Supporting Information is available for this article:

Fig. S1 Geographic location of the 43 provenances sampled.

Fig. S2 Schematic representation of the tree-ring traits examined in this study.

Fig. S3 Monthly variation of total precipitation **(**MAP; **a)**, maximum temperature **(**Tmax; **b)** and soil moisture index **(**SMI; **c)** for the 1996-2008 period.

Fig. S4 Growth performance of white spruce seed provenances at the common garden site.

Fig. S5 Provenance means for basal area increment (BAI) plotted against mean annual temperature (MAT; **a**), summer soil moisture index (Summer_SMI; **b**), and annual number of dry days (ADD; **c**) at provenance origin.

Fig. S6 Impact of drought on cell morphology.

Fig. S7 Box plots for growth recovery **(a)** and growth resilience **(b)** for the 2001-2002 drought event.

Fig. S8 Relationship between radial growth (mean BAI for each provenance) and growth recovery **(a)**, growth relative resilience **(b)**, and growth resilience **(c)**.

Table S1 Mean annual bioclimatic characteristics of the 43 provenances and the common garden site (indicated in italics) over the 1950-1980 period.

Table S2 Basic statistics for the studied wood traits.

Table S3 Basic statistics estimated per SNP for the 6,386 SNPs used in the present study. See

separate file.

Table S4 Linear modeling analysis for long-term **(a)** and the 2002 **(b)** drought-resilience (DR) traits.

Table S5 Pairwise Pearson correlations between the studied traits.

Methods S1 Calculation of the competition index and detrending methods.

Notes S1 Relationship between the drought-resilience traits (i.e. Rc_{2002}, Rl_{2002} and Rr_{2002}) and the climatic variables at provenance origins.

Fig. S1 Geographic location of the 43 provenances sampled. The natural distribution of white spruce in eastern Canada is represented in green. The red rectangle marks the study area (as presented in Figure 4a, b). The geographical origins of sampled provenances are indicated by black circles and the location of the common garden site is indicated by a red star.

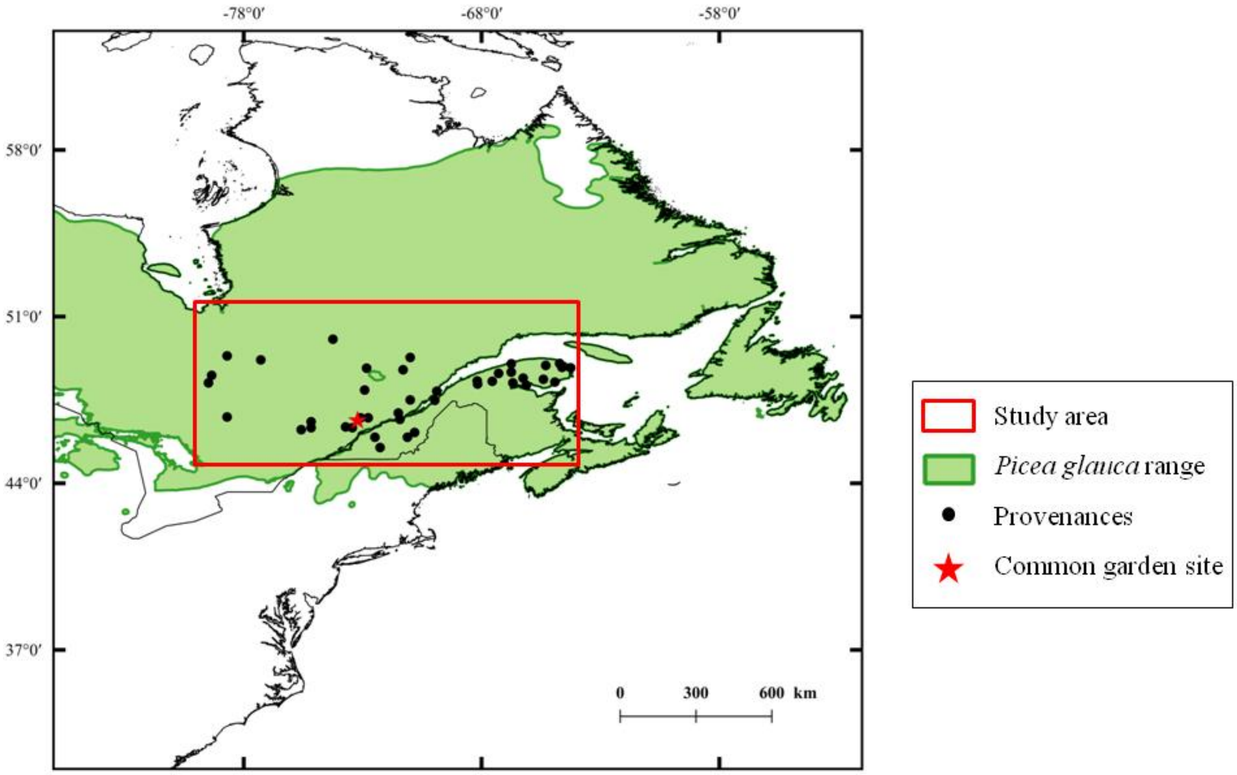

Fig. S2 Schematic representation of the tree-ring traits examined in this study.

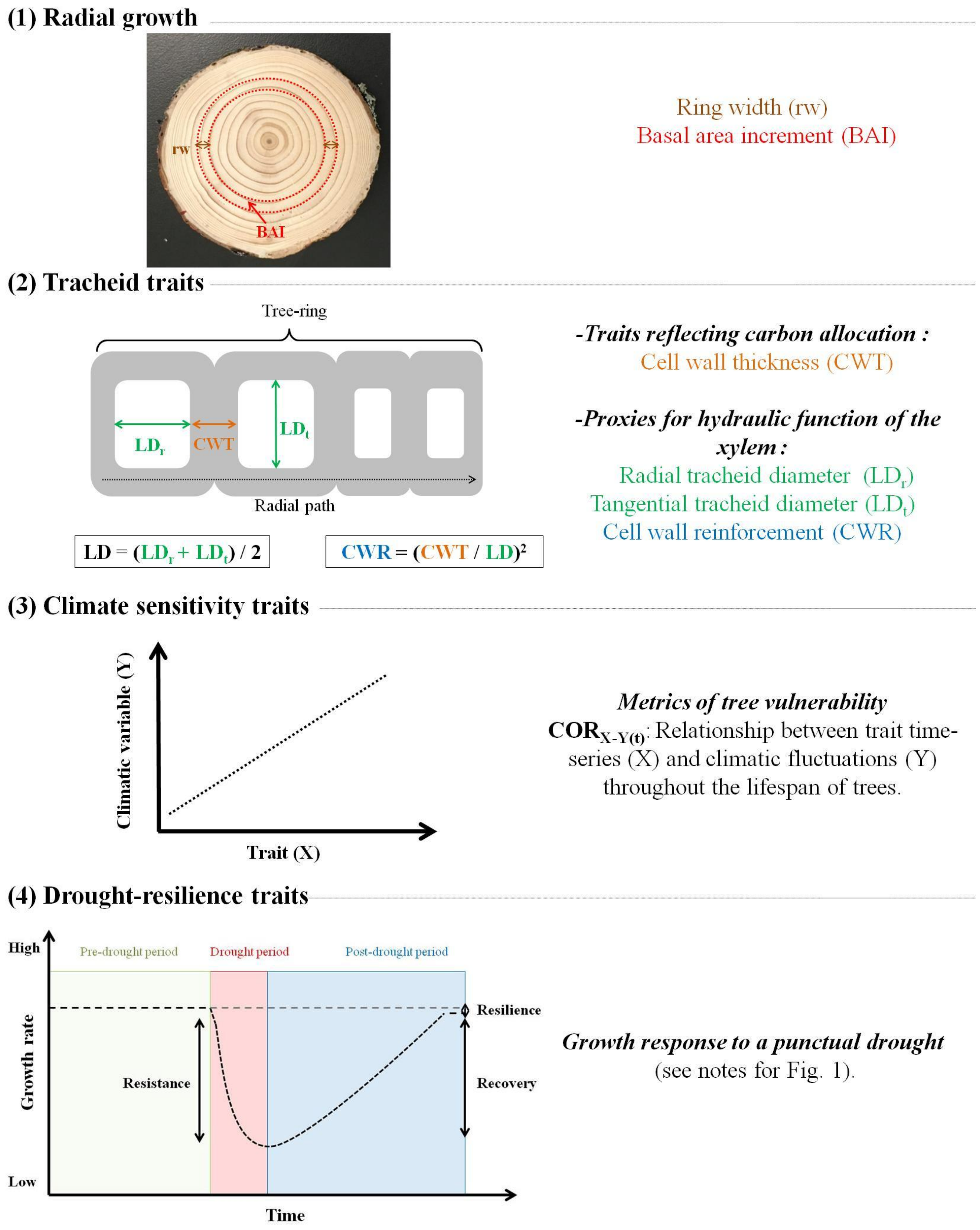

Fig. S3 Monthly variation of total precipitation **(**MAP; **a)**, maximum temperature **(**Tmax; **b)** and soil moisture index **(**SMI; **c)** for the 1996-2008 period. Coloured lines represent dry years.

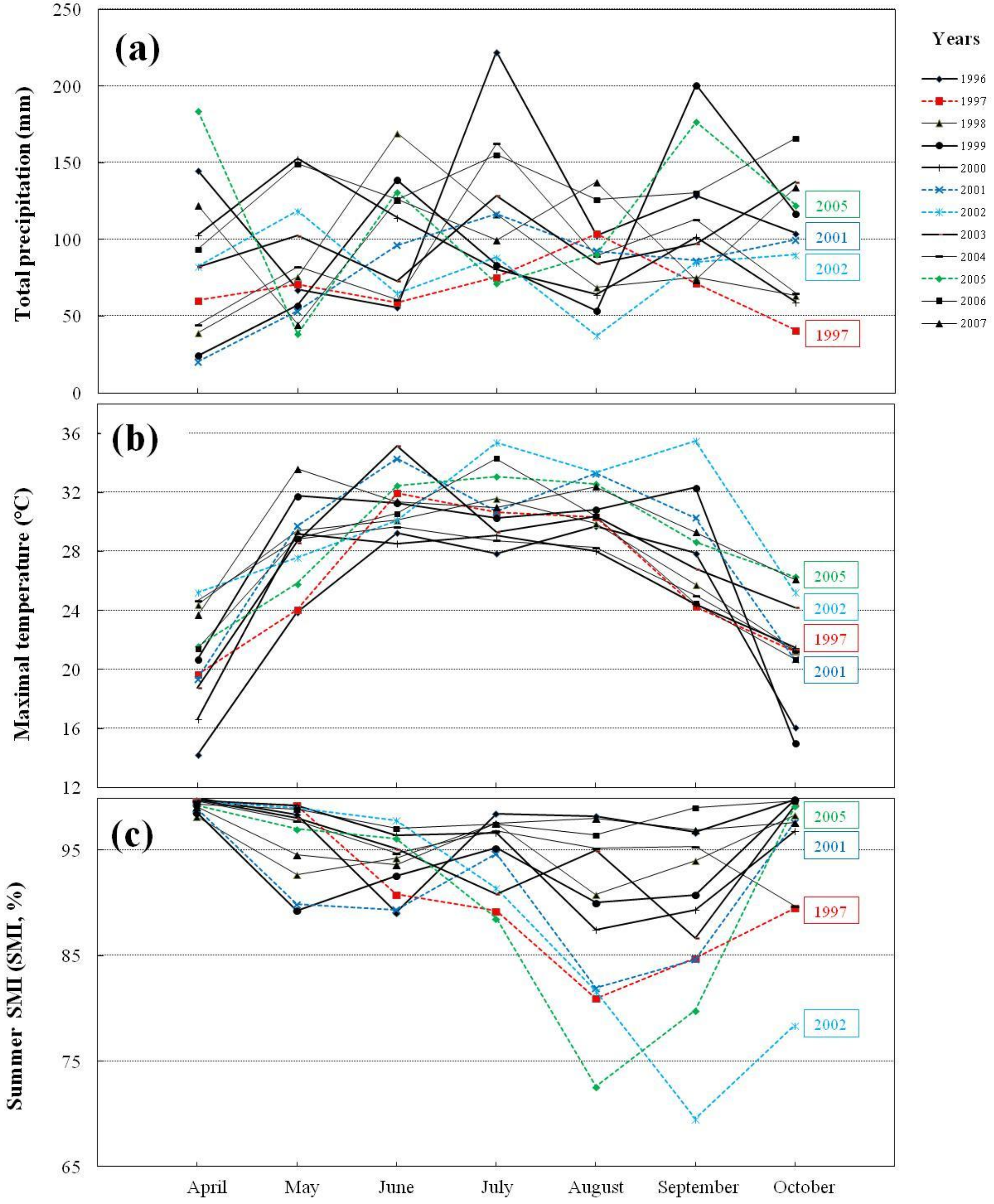

Fig. S4 Growth performance of white spruce seed provenances at the common garden site. The box portion of the box plot is defined by two lines at the 25th percentile and 75th percentile. The median is represented by the intermediate line within the box. The whiskers indicate the variability outside the upper and lower quartiles. Dots represent outliers, i.e. values >1.5 times and <3 times the interquantile range beyond either end of the box. The best performing provenance (POP_42) is shown in green, while the worst performing provenance is shown in grey (POP_25).

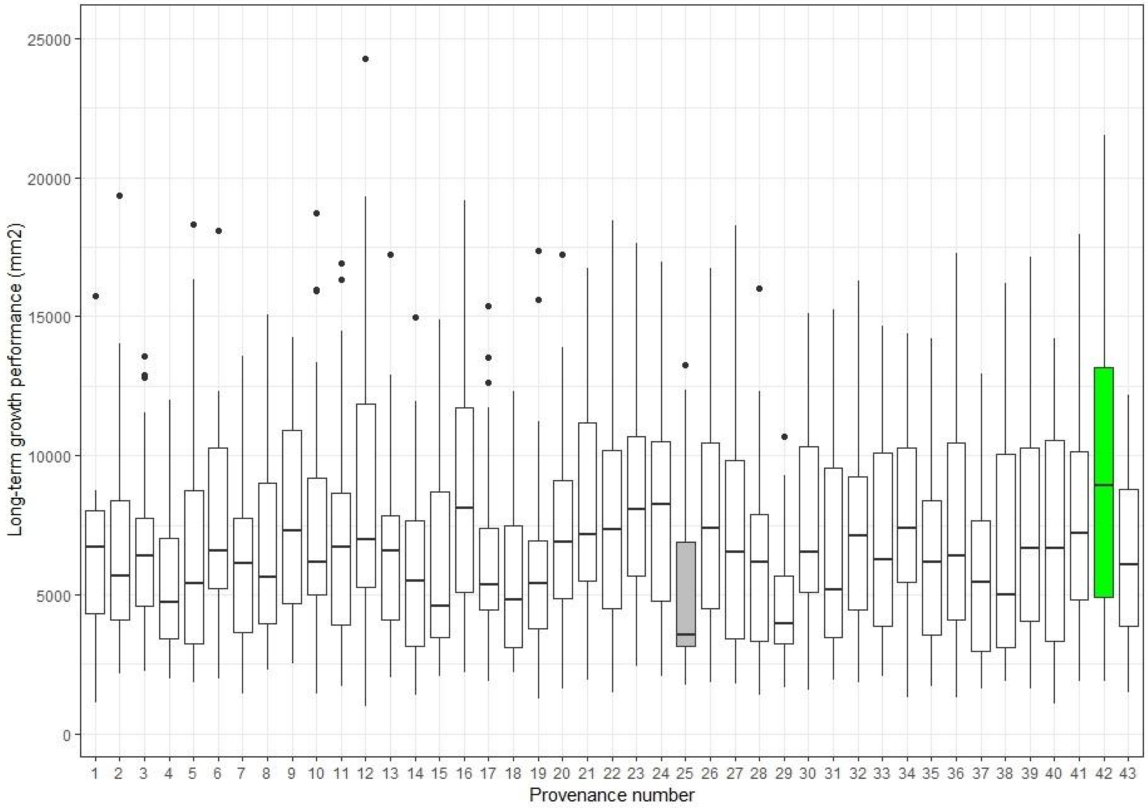

Fig. S5 Provenance means for basal area increment (BAI) plotted against mean annual temperature (MAT; **a**), summer soil moisture index (Summer_SMI; **b**), and annual number of dry days (ADD; **c**) at provenance origin. Curvilinear regressions were performed to study the relationship between long-term growth performance (i.e. observed values for BAI, as reported in Table S3) and MAT **(a)**, Summer_SMI **(b),** and ADD **(c)** recorded at the seed origin of sampled provenances. Values of MAT, Summer_SMI, and ADD for the common garden site are enclosed by a dashed green line. After testing several polynomial regression equations, the best model was selected based on both AIC and AICc criteria (detailed procedure described at https://rcompanion.org/rcompanion/e_03.html).

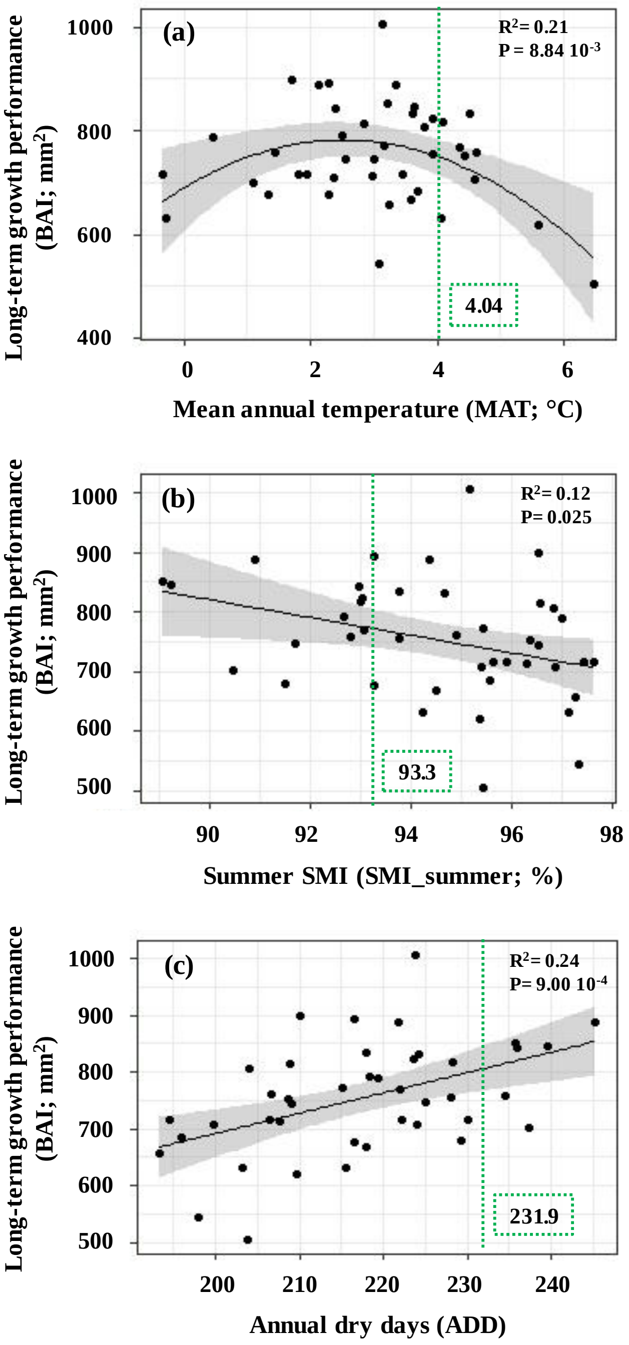

Fig. S6 Impact of drought on cell morphology. Correlations between SMI and radial lumen diameter **(a)**, cell wall thickness **(b),** radial cell wall reinforcement **(c)** and wood density **(d)** are presented**.** For each provenance, the correlations between the mean residual chronology and SMI are presented for May to October of the previous (t-1) growing season and for the current growing season (t), for the 1989-2007 period. The scale bar reports positive (red) and negative (blue) correlation coefficients, while significant relationships ($P < 0.05$) are indicated by dots in the correlation matrices. Drought resulted in the formation of tracheids with smaller lumen diameters for most of the provenances surveyed **(b)**. Radial cell wall reinforcement and wood density were significantly and negatively correlated to SMI for some provenances in $Jul_{(t)}$ **(c, d)**.

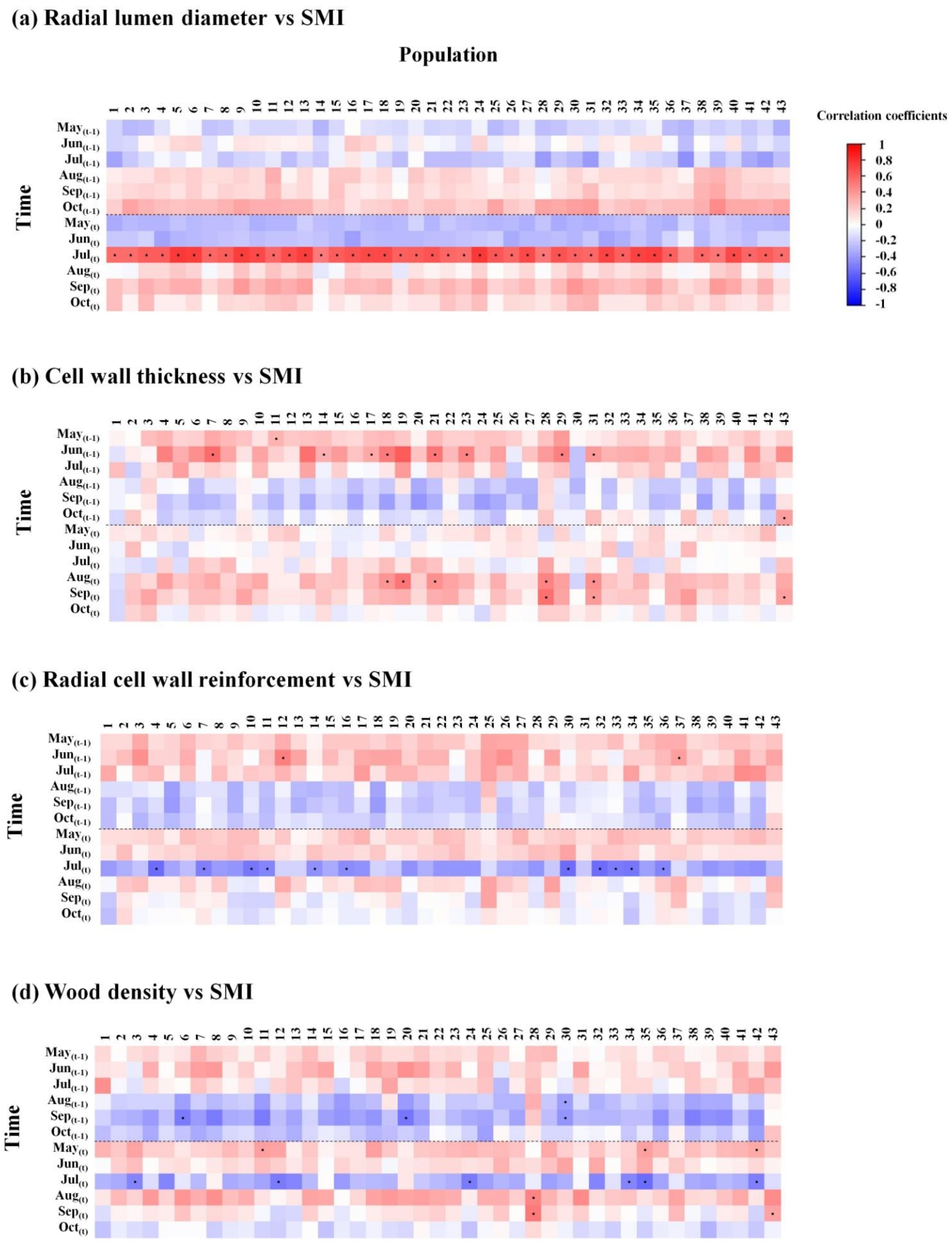
(a) Radial lumen diameter vs SMI
Population
Correlation coefficients
1
0.8
0.6
0.4
0.2
0
-0.2
-0.4
-0.6
-0.8
-1
Time
May(t-1)
Jun(t-1)
Jul(t-1)
Aug(t-1)
Sep(t-1)
Oct(t-1)
May(t)
Jun(t)
Jul(t)
Aug(t)
Sep(t)
Oct(t)
(b) Cell wall thickness vs SMI
(c) Radial cell wall reinforcement vs SMI
(d) Wood density vs SMI

Fig. S7 Box plots for growth recovery **(a)** and growth resilience **(b)** for the 2001-2002 drought event. The box portion of the box plot is defined by two lines at the 25th and 75th percentile. The median is represented by the intermediate line within the box. The whiskers indicate the variability outside the upper and lower quartiles. Dots represent outliers, i.e. values >1.5 times and <3 times the interquantile range beyond either end of the box. In each panel, the provenance with the highest mean for the trait tested is indicated in green. The two provenances exhibiting the lowest Rc_{2002} values are indicated in grey **(a)**. For growth resilience, the extreme provenances identified for the Summer_SMI gradient are presented in orange (i.e. POP_7 and POP_37 with the lowest predicted resilience values, please refer to **Fig. 4c**) and red (i.e. POP_34 with the highest predicted resilience value) (**b**). The two southern provenances (i.e. POP_25 and POP_43, see **Fig. 4c**) are indicated in blue.

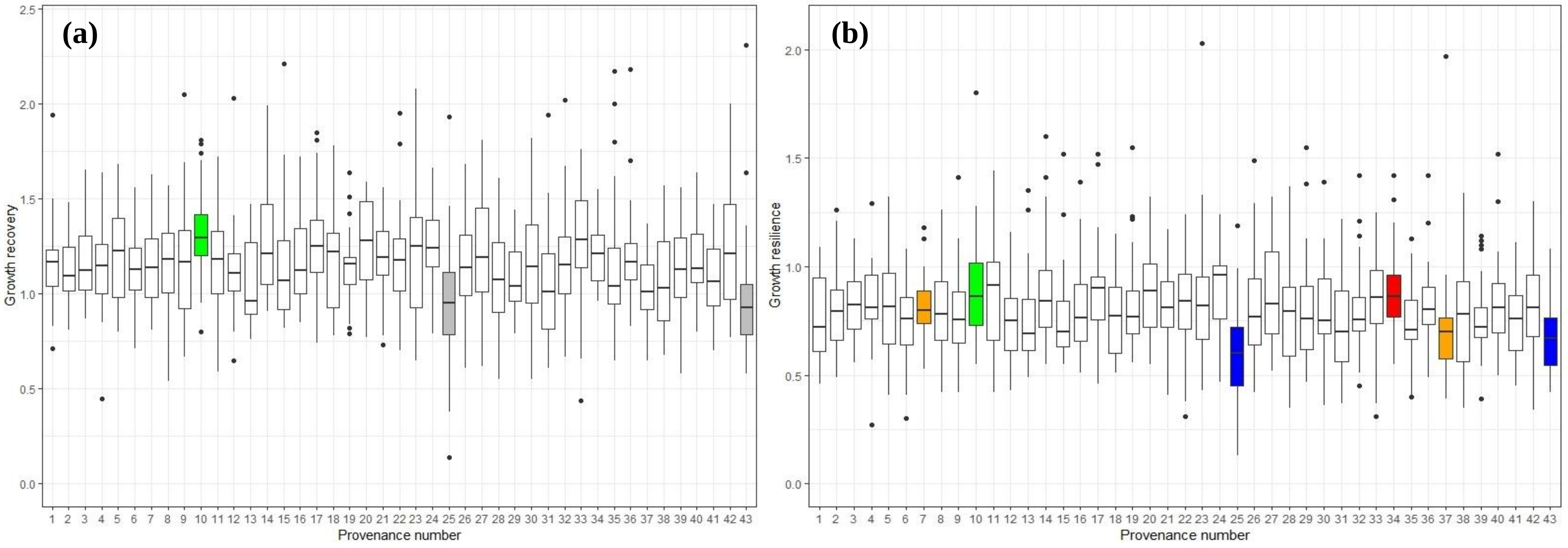

Fig. S8 Relationship between radial growth (mean BAI for each provenance) and growth recovery **(a)**, growth relative resilience **(b)**, and growth resilience **(c)**. Correlation analyses were performed as described for **Figure S5**.

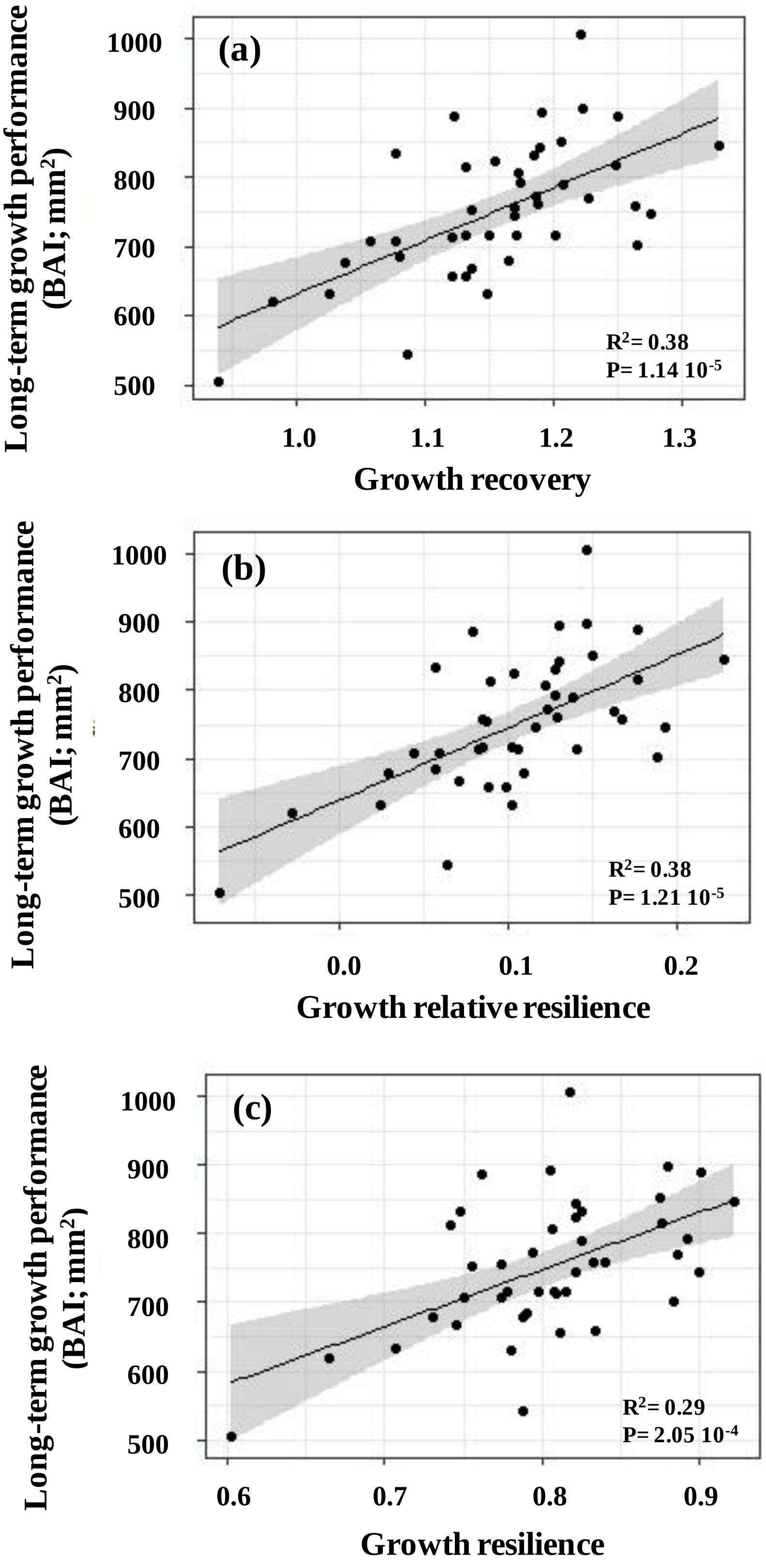

New Phytologist

Table S1 Mean annual bioclimatic characteristics of the 43 provenances and the common garden site (indicated in italics) over the 1950-1980 period. Locations are ordered by averages of mean annual precipitation, MAP. MAT: mean annual temperature; MAP: mean annual precipitation; FD: mean annual number of frost days; ADD: mean annual number of dry days.

Provenance or site name	Provenance number or site	Latitude	Longitude	Elevation	MAT (°C)	MAP (mm)	FD (days)	ADD (days)	Summer_SMI (%)
**Parc des Laurentides**	**36**	47.46	-71.16	732	0.47	1322.15	221.32	219.42	96.99
**Parc des Laurentides**	**37**	47.46	-71.16	792	-0.29	1242.59	231.29	215.55	97.11
**Valcartier**	**35**	46.95	-71.5	152	3.57	1240.85	184.06	217.94	94.51
**Racine**	**43**	45.5	-72.27	244	5.6	1191.46	162.35	209.71	95.38
**Lampton**	**39**	45.93	-71.12	305	4.42	1167.91	176.94	208.71	96.35
**St-Nicolas**	**40**	46.68	-71.43	91	4.63	1151.91	168.26	206.74	94.88
**St-Emile de l'Energie**	**42**	46.37	-73.72	396	3.13	1142.18	198.55	223.81	95.15
**St-Damien de Brandon**	**41**	46.33	-73.43	183	4.52	1128.13	177.13	218.03	93.77
**Canton Boyer**	**9**	46.58	-75.17	243	3.92	1094.13	184.52	228.13	93.77
**Cap des Rosiers**	**1**	48.85	-64.25	15	3.45	1093.96	177.68	194.48	97.44
**Drummondville**	**25**	45.93	-72.48	61	6.48	1092.85	148.84	203.9	95.43
**Canton Lesage**	**24**	46.33	-75.17	259	3.36	1085.94	203.35	245.26	90.91
**Beauceville**	**38**	46.13	-70.82	213	4.6	1081.81	176.87	224.03	95.4
Mastigouche	***site***	*46.63*	*-73.22*	*230*	4.04	1064.86	185.97	231.97	93.28
**Baie Gaspé Sud**	**2**	48.87	-64.62	91	2.98	1064.44	190.84	207.74	96.28
**Canton Port-Daniel**	**19**	48.25	-64.92	170	3.23	1062.12	179.71	193.35	97.26
**Canton Port-Daniel**	**4**	48.25	-64.92	170	3.25	1061.97	179.77	193.32	97.25
**Canton Desaulniers**	**22**	46.75	-73.08	365	3.62	1047.5	186.16	224.16	94.68
**Carleton**	**30**	48.12	-66.12	61	3.79	1041.37	174.81	203.97	96.84
**Canton Garin**	**6**	48.37	-65.4	243	2.85	1031.63	189.13	208.84	96.57
**St-Roch de Mékinac**	**26**	46.75	-72.77	152	3.94	1030.66	180.87	223.65	93.03
**Canton Sydenham**	**13**	49.03	-64.72	251	2.36	1030.54	193.45	199.81	96.86
**Canton Clapperton**	**7**	48.42	-66.25	487	1.94	1018.35	196.97	206.39	97.64
**Canton Boutet**	**5**	48.67	-66.75	335	1.82	1013.93	210.97	230.03	95.62
**Canton McGill**	**20**	46.25	-75.58	304	4.09	1004.29	182.03	228.16	93
**Canton Vallée**	**3**	48.22	-66.68	426	2.56	996.68	191.65	209	96.51
**Canton Ouimet**	**18**	48.3	-68.18	320	4.06	986.81	171.65	203.16	94.24
**Canton Holland**	**23**	48.95	-65.3	441	1.7	981.45	201.52	210.03	96.52
**Canton Hébécourt**	**16**	48.53	-79.3	224	2.29	947.83	190.45	216.45	93.28
**Lac Humqui**	**31**	48.28	-67.55	244	2.29	947.83	190.45	216.45	93.28
**Canton Richardson**	**8**	50.05	-74.25	525	-0.34	917.68	215.81	222.29	95.9
**Canton Blais**	**17**	48.62	-67.28	167	3.01	910.61	192.29	225.03	91.71
**Canton Chaumonot**	**21**	47.92	-72.92	274	2.4	905.98	197	236	92.96
**St-Siméon**	**32**	47.87	-69.88	213	3.17	901.42	178.19	215.13	95.42
**Grand Métis**	**28**	48.63	-68.17	61	3.7	900.42	168.84	196.03	95.58
**Canton Dasserat**	**11**	48.22	-79.48	289	2.5	888.12	188.94	218.35	92.68
**Lac à la l'Ours**	**33**	48.77	-71.3	335	1.44	887.04	206.74	234.58	92.81
**Canton Cimon**	**12**	49.28	-71,00	198	2.12	877.32	188.9	221.77	94.35
**Canton Booth**	**10**	46.78	-78.7	365	3.63	844.68	185.03	239.61	89.25
**Canton Fraser**	**14**	49.18	-77.28	289	1.09	840.42	214.65	237.29	90.47
**Kamouraska**	**27**	47.48	-69.97	30	4.35	839.55	166.84	221.97	93.07
**Cap Chat**	**29**	49.17	-66.75	30	3.09	834.65	173.13	198.03	97.33
**Canton Bacon**	**15**	49.35	-78.7	289	1.33	812.26	209.29	229.19	91.52
**Parc Chibougamau**	**34**	48.83	-72.83	244	3.21	789.17	185.61	235.61	89.07

Table S2 Basic statistics for the studied wood traits. For each provenance, the number of trees, the number of families and the observed values (means and standard error) are presented. Provenance means are the average annual values for the 1995-2005 period. Statistical significance levels of the one-way ANOVAs (provenance) are reported in the last row, * P<0.05, ** P<0.01, and *** P<0.001.

Provenance number	No. Families	No. Sampled trees	BAI (mm^2)	WD ($kg \cdot m^{-3}$)	CWT (µm)	LDr (µm)	LD (µm)	CWRr ($x10^{-2}$)	CWR ($x10^{-2}$)
1	4	18	714.61 (85.41)	435.54 (7.53)	4.21 (0.08)	24.02 (0.44)	23.11 (0.37)	3.31 (0.17)	3.53 (0.16)
2	5	40	713.87 (57.29)	431.48 (5.05)	4.21 (0.05)	24.11 (0.30)	23.39 (0.25)	3.17 (0.12)	3.35 (0.11)
3	4	31	744.86 (65.08)	431.85 (5.73)	4.20 (0.06)	24.53 (0.34)	23.36 (0.28)	3.04 (0.13)	3.37 (0.12)
4	4	26	657.62 (71.06)	428.08 (6.26)	4.17 (0.07)	24.05 (0.37)	23.34 (0.31)	3.34 (0.15)	3.43 (0.13)
5	5	38	714.82 (58.78)	421.24 (5.18)	4.10 (0.06)	24.54 (0.30)	23.49 (0.25)	2.92 (0.12)	3.17 (0.11)
6	5	22	813.53 (77.25)	439.48 (6.81)	4.32 (0.07)	24.39 (0.40)	23.36 (0.33)	3.41 (0.16)	3.65 (0.14)
7	5	34	715.43 (62.14)	427.93 (5.48)	4.12 (0.06)	24.29 (0.32)	23.09 (0.27)	3.00 (0.13)	3.31 (0.12)
8	5	35	716.35 (61.25)	423.09 (5.40)	4.13 (0.06)	24.17 (0.32)	23.50 (0.26)	3.05 (0.13)	3.21 (0.11)
9	2	34	756.25 (62.14)	417.98 (5.48)	4.15 (0.06)	24.93 (0.32)	24.04 (0.27)	2.91 (0.13)	3.12 (0.12)
10	4	36	845.19 (60.39)	420.78 (5.32)	4.04 (0.06)	23.86 (0.31)	23.11 (0.26)	3.01 (0.12)	3.18 (0.11)
11	3	40	791.81 (57.29)	407.11 (5.05)	4.00 (0.05)	25.44 (0.30)	23.93 (0.25)	2.59 (0.12)	2.92 (0.11)
12	5	25	887.30 (72.47)	420.35 (6.39)	4.22 (0.07)	25.05 (0.38)	24.20 (0.31)	2.96 (0.15)	3.17 (0.14)
13	1	34	708.09 (62.14)	416.68 (5.48)	4.09 (0.06)	25.69 (0.32)	23.82 (0.27)	2.62 (0.13)	3.10 (0.12)
14	5	33	701.51 (63.08)	408.97 (5.56)	4.01 (0.06)	25.05 (0.33)	23.82 (0.27)	2.71 (0.13)	2.97 (0.12)
15	5	22	678.71 (77.25)	422.49 (6.81)	4.18 (0.07)	25.42 (0.40)	23.90 (0.33)	2.82 (0.16)	3.19 (0.14)
16	4	45	893.23 (54.02)	409.63 (4.76)	4.10 (0.05)	25.39 (0.28)	24.38 (0.23)	2.71 (0.11)	2.93 (0.10)
17	4	25	745.15 (72.47)	429.07 (6.39)	4.18 (0.07)	24.77 (0.38)	23.44 (0.31)	3.06 (0.15)	3.40 (0.14)
18	5	28	630.84 (68.48)	432.90 (6.03)	4.10 (0.06)	23.61 (0.35)	22.71 (0.30)	3.11 (0.14)	3.37 (0.13)
19	5	45	657.24 (54.02)	414.30 (4.76)	3.93 (0.05)	23.91 (0.28)	22.96 (0.23)	2.83 (0.11)	3.05 (0.10)
20	5	28	816.04 (68.48)	415.86 (6.03)	4.08 (0.06)	24.16 (0.35)	23.74 (0.30)	2.98 (0.14)	3.07 (0.13)
21	5	41	842.36 (56.59)	436.97 (4.99)	4.40 (0.05)	24.79 (0.29)	24.01 (0.24)	3.29 (0.12)	3.49 (0.11)
22	5	41	831.65 (56.59)	421.54 (4.99)	4.08 (0.05)	24.09 (0.29)	23.28 (0.24)	2.98 (0.12)	3.19 (0.11)
23	3	25	898.04 (72.47)	411.65 (6.39)	4.11 (0.07)	25.18 (0.38)	24.27 (0.31)	2.76 (0.15)	2.97 (0.14)
24	5	35	888.55 (61.25)	396.25 (5.40)	3.96 (0.06)	25.83 (0.32)	24.51 (0.26)	2.44 (0.13)	2.72 (0.11)
25	5	36	504.09 (60.39)	463.89 (5.32)	4.40 (0.06)	22.49 (0.31)	22.14 (0.26)	4.10 (0.12)	4.17 (0.11)
26	5	40	823.72 (57.29)	410.77 (5.05)	4.03 (0.05)	24.99 (0.30)	23.90 (0.25)	2.73 (0.12)	2.96 (0.11)
27	5	37	769.46 (59.57)	412.16 (5.25)	3.96 (0.06)	24.30 (0.31)	23.35 (0.26)	2.78 (0.12)	2.99 (0.11)
28	5	31	683.53 (65.08)	421.04 (5.73)	4.15 (0.06)	23.87 (0.34)	23.73 (0.28)	3.17 (0.13)	3.18 (0.12)
29	5	41	542.80 (56.59)	434.54 (4.99)	4.11 (0.05)	23.40 (0.29)	22.57 (0.24)	3.24 (0.12)	3.46 (0.11)
30	5	37	805.94 (59.57)	418.98 (5.25)	4.10 (0.06)	24.80 (0.31)	23.70 (0.26)	2.87 (0.12)	3.13 (0.11)
31	5	36	677.34 (60.39)	412.23 (5.32)	4.06 (0.06)	24.67 (0.31)	23.88 (0.26)	2.83 (0.12)	3.01 (0.11)
32	5	38	773.01 (58.78)	406.80 (5.18)	3.94 (0.06)	24.49 (0.30)	23.56 (0.25)	2.71 (0.12)	2.91 (0.11)
33	5	36	757.02 (60.39)	411.61 (5.32)	4.07 (0.06)	24.72 (0.31)	23.92 (0.26)	2.79 (0.12)	2.98 (0.11)
34	5	38	851.25 (58.78)	394.06 (5.18)	3.86 (0.06)	25.15 (0.30)	24.10 (0.25)	2.47 (0.12)	2.68 (0.11)
35	5	40	667.54 (57.29)	420.57 (5.05)	4.19 (0.05)	24.45 (0.30)	24.02 (0.25)	3.07 (0.12)	3.16 (0.11)
36	5	35	788.64 (61.25)	408.13 (5.40)	3.91 (0.06)	24.15 (0.32)	23.34 (0.26)	2.74 (0.13)	2.91 (0.11)
37	5	36	632.03 (60.39)	400.24 (5.32)	3.76 (0.06)	23.71 (0.31)	22.89 (0.26)	2.65 (0.12)	2.83 (0.11)
38	5	34	706.45 (62.14)	414.60 (5.48)	4.04 (0.06)	24.40 (0.32)	23.63 (0.27)	2.85 (0.13)	3.03 (0.12)
39	5	37	753.21 (59.57)	403.70 (5.25)	3.97 (0.06)	24.62 (0.31)	23.97 (0.26)	2.74 (0.12)	2.87 (0.11)
40	5	38	759.31 (58.78)	410.00 (5.18)	4.03 (0.06)	24.64 (0.30)	23.89 (0.25)	2.77 (0.12)	2.93 (0.11)
41	5	37	832.87 (59.57)	408.43 (5.25)	4.00 (0.06)	24.85 (0.31)	23.85 (0.26)	2.69 (0.12)	2.92 (0.11)
42	5	37	1006.50 [illegible]	410.20 (5.25)	4.09 (0.06)	25.31 (0.31)	24.24 (0.26)	2.74 (0.12)	2.97 (0.11)
43	4	36	619.25 (60.39)	410.91 (5.32)	4.00 (0.06)	24.40 (0.31)	23.62 (0.26)	2.82 (0.12)	2.98 (0.11)
P-value	-	-	***	***	***	***	***	***	***

Table S3 Basic statistics estimated per SNP for the 6,386 SNPs used in the present study. Observed heterozygosity (H_O), genetic diversity (H_S), overall gene diversity (H_T), gene diversity among samples (D_{ST}) and the population differentiation due to genetic structure (F_{ST}) per loci were assessed following the classical method of Nei. The 6,386 validated SNPs of the PGAS1 microarray are presented. See separate file.

Table S4 Linear modeling analysis for long-term **(a)** and the 2002 **(b)** drought-resilience (DR) traits. The block and tree size effects are fixed. The tree size effect refers to the sum of basal area increment in 2002 for drought-resilience (DR) traits evaluated for the major drought of that year **(a)**. For long-term DR traits **(b),** tree size refers to the average of basal area increments measured at 1997, 2002 and 2005. The terms pop and fam(pop) refer to the provenance and the family nested in the provenance random effects, respectively. The family-by-block interaction was also included as a random effect in our models. Likelihood ratio tests were performed in ASReml-R (lrt.asreml function) to determine the significance of each random effect by comparing the full model with a reduced model without the random effect to be tested. For random effects, *P-values* of the likelihood ratio test are reported. For fixed effects, WALD statistics and *P-values* with their corresponding levels of significance are reported. *P<0.05, **P<0.01, ***P<0.001.

(a)

		Rs_{2002}		Rc_{2002}		Rl_{2002}		Rr_{2002}	
**Effect**	**Type**	**WALD**	***P-value***	**WALD**	***P-value***	**WALD**	***P-value***	**WALD**	***P-value***
**intercept**	-	34435	<0.001 ***	478.67	<0.001 ***	6234.9	<0.001 ***	130.867	<0.001 ***
**block**	fixed	30	<0.001 ***	19.4	0.0016 **	51.1	<0.001 ***	26.862	<0.001 ***
**tree size**	fixed	23	<0.001 ***	28.4	<0.001 ***	71.5	<0.001 ***	40.732	<0.001 ***
**pop**	random	-	0.4975	-	<0.001 ***	-	<0.001 ***	-	<0.001 ***
**fam(pop)**	random	-	0.0318 *	-	<0.001 ***	-	<0.001 ***	-	<0.001 ***
**fam:block**	random	-	<0.001 ***	-	0.4088	-	0.0012 **	-	0.1932

(b)

		Rs_{MEAN}		Rc_{MEAN}		Rl_{MEAN}		Rr_{MEAN}	
**Effect**	**Type**	**WALD**	***P-value***	**WALD**	***P-value***	**WALD**	***P-value***	**WALD**	***P-value***
**intercept**	-	40434	<0.001 ***	19659.5	<0.001 ***	19477.3	<0.001 ***	1524.66	<0.001 ***
**block**	fixed	1	0.2475	3.7	0.0547	7.5	<0.001 ***	6.29	0.0122 *
**tree size**	fixed	29	<0.001 ***	24.8	<0.001 ***	66.0	<0.001 ***	44.41	<0.001 ***
**pop**	random	-	0.1063	-	<0.001 ***	-	<0.001 ***	-	<0.001 ***
**fam(pop)**	random	-	0.3102	-	0.0968	-	0.4975	-	0.1619
**fam:block**	random	-	0.3948	-	0.5	-	0.1936	-	0.5

New Phytologist

Table S5 Pairwise Pearson correlations between the studied traits. Pearson correlations were performed using provenance means of the drought-resilience and wood traits, using the Hmisc and PerformanceAnalytics R packages.

	BAI	Rs_{2002}	Rc_{2002}	Rl_{2002}	Rr_{2002}	Rs_{MEAN}	Rc_{MEAN}	Rl_{MEAN}	Rr_{MEAN}	WD	CWT	LDr	LD	CWRr	CWR	$COR_{LDr-july(t)}$	$COR_{CWRr-july(t)}$
BAI	1.00	-0,03	**0,62**	**0,54**	**0,61**	-0,30	**0,58**	0,27	**0,51**	**-0,45**	-0,10	**0,65**	**0,69**	**-0,48**	**-0,46**	0,08	-0,37
Rs_{2002}		1.00	-0,01	**0,43**	0,07	**0,54**	0,15	**0,45**	0,22	-0,18	-0,22	0,09	0,02	-0,19	-0,17	0,04	-0,17
Rc_{2002}			1.00	**0,89**	**0,99**	-0,20	**0,82**	**0,49**	**0,72**	-0,29	-0,13	**0,38**	**0,35**	**-0,34**	**-0,31**	0,11	**-0,48**
Rl_{2002}				1.00	**0,93**	0,07	**0,82**	**0,67**	**0,78**	**-0,36**	-0,24	**0,39**	**0,33**	**-0,42**	**-0,38**	0,12	**-0,52**
Rr_{2002}					1.00	-0,16	**0,85**	**0,55**	**0,77**	**-0,33**	-0,19	**0,39**	**0,34**	**-0,39**	**-0,35**	0,10	**-0,48**
Rs_{MEAN}						1.00	-0,24	**0,53**	0,01	0,05	-0,10	-0,09	-0,23	-0,04	0,03	-0,15	0,04
Rc_{MEAN}							1.00	**0,63**	**0,92**	**-0,37**	-0,26	**0,31**	**0,32**	**-0,36**	**-0,36**	-0,01	**-0,44**
Rl_{MEAN}								1.00	**0,84**	**-0,39**	**-0,43**	0,21	0,10	**-0,44**	**-0,39**	-0,25	-0,30
Rr_{MEAN}									1.00	**-0,45**	**-0,40**	**0,32**	0,26	**-0,48**	**-0,45**	-0,17	**-0,38**
WD										1.00	**0,85**	**-0,59**	**-0,62**	**0,94**	**0,99**	0,17	**0,36**
CWT											1.00	-0,18	-0,12	**0,79**	**0,84**	**0,36**	0,27
LDr												1.00	**0,88**	**-0,72**	**-0,60**	0,21	-0,21
LD													1.00	**-0,63**	**-0,63**	0,23	-0,29
CWRr														1.00	**0,96**	0,11	0,30
CWR															1.00	0,15	**0,37**
$COR_{LDr-july(t)}$																1.00	-0,01
$COR_{CWRr-july(t)}$																	1.00

Methods S1 Calculation of the competition index and detrending methods.

Considering the relatively high plantation density of the common garden site, we hypothesised that competition might have had a significant impact on tree growth. A competition index (hci) was therefore calculated for each tree based on tree height in 1997 (Braathe, 1980) as follows:

$$\text{hci}= \sum^{n}_{i=1} H_i/(H_j \text{ x } dist_{i.j}) \qquad (1)$$

where n is the number of competing trees (up to 8), H_i is the height of ith neighbouring tree, H_j is the height of jth focal tree and $dist_{i.j}$ is the distance between neighbouring trees and tree *j*. This competition index was assumed to be representative of the studied period. Since commercial thinning was carried out at the site during the summer of 1997, an increase in radial growth was observed in subsequent years (See **Figure 2** for details).

With the objective of detecting climate signals for annual measurements of basal area increment (BAI), wood density (WD), cell wall thickness (CWT), radial lumen diameter (LDr) and radial cell wall reinforcement (CWRr), data were detrended by fitting a GAMM model as follows:

$$\text{Trait}_{it} \sim 1 + s(\text{cambial age}_t) + s(\text{cambial age}_t, \text{tree\_id}) + \text{hci}_i + \text{BA}_{i\ +} e_{it} \qquad (2)$$

where *i* represents the tree and *t* represents the year. Trait is the quantitative trait, cambial age is an estimation of tree age based on the ring, tree_id is the tree, hci refers to the competition index, BA istree size (i.e sum of basal area increments at the time of sampling) and e_{it} is the error term. Smoothing terms were represented using default package settings, and are indicated by *s* in the model. The term *s*(cambial age$_t$, tree_id) refers to a random smooth by tree_id (i.e. a separate smooth for each of the 1,481 trees). The term hci was kept in the model for the basal area increment only.

Trait values were transformed when necessary to improve the normality of the data (see Table below). Both normality and homoscedasticity of the residuals were visually checked. In addition, Shapiro tests were performed to inspect the normality of the residuals for individual provenance sets.

Trait name	Transformation
BAI	Square root
WD	Log
LDr	No transformation
CWT	Log
CWRr	Log

References

Braathe P. 1980. Height increment of young single trees in relation to height and distance of neighbouring trees. *Mitteilungen der Forstl. Bundesversuchsanstalt* **130:** 43-48.

Notes S1 Relationships between drought-resilience traits (i.e. Rc_{2002}. Rl_{2002} and Rr_{2002}) and the climate variables at provenance origins. Four predictors were tested for all traits: mean annual temperature (MAT), soil moisture index in summer (Summer_SMI), mean annual dry days (ADD) and mean annual precipitation (MAP). For Rc_{2002} and Rr_{2002}, the results using only MAT and Summer_SMI as potential predictors are also presented.

R command line used for MARS analyses:

```
> earth_model <- earth (trait~ .. data = data.earth. nfold=10. penalty = 2. thresh = 0.001. minspan = 0. endspan = 0. newvar.penalty = 0. fast.k = 20. fast.beta = 1)
```

1.1. Detailed MARS analyses for growth resilience, Rl_{2002}

Predictors used in this analysis: MAT, Summer_SMI, ADD and MAP.

- Equation retained by the earth model:

Rl_{2002}= 0.79764- 0.067265 * pmax(0, MAT - 3.9419) + 0.021845* pmax(0, 94.354 - Summer_SMI)

- Other summary details:

Selected 3 of 15 terms, and 2 of 4 predictors
Termination condition: Reached nk 21
Importance: MAT, Summer_SMI, ...
Number of terms at each degree of interaction: 1 2 (additive model)
GCV 0.0021792 RSS 0.073181 GRSq 0.47543 RSq 0.57059 CVRSq 0.040576

Note: the cross-validation sd's below are standard deviations across folds

Cross validation: nterms 3.60 sd 1.17 nvars 1.90 sd 0.32

CVRSq	sd	MaxErr	sd
0.041	0.578	-0.189	0.0869

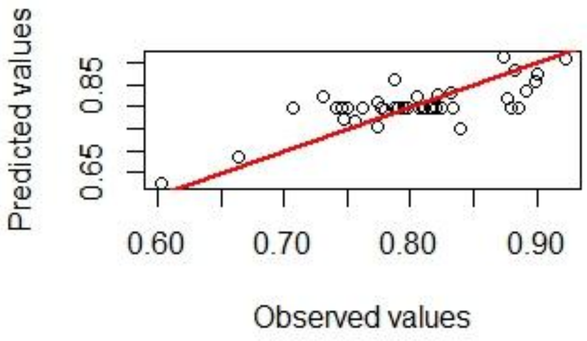

Figure 1.1: Observed values plotted against the predicted values of *Rl*.

1.2. Detailed MARS analyses for growth recovery, Rc_{2002}

1.2.1. Using MAT, Summer_SMI, ADD and MAP as predictors

- Equation retained by the earth model:

Rc_{2002}= 1.137- 0.074457 * pmax(0, MAT - 3.9419) + 0.0050129 * pmax(0, 215.13 - ADD)

- Other summary details:

Selected 3 of 15 terms, and 2 of 4 predictors
Termination condition: Reached nk 21
Importance: ADD, MAT, ...
Number of terms at each degree of interaction: 1 2 (additive model)
GCV 0.0033123 RSS 0.11123 GRSq 0.48009 RSq 0.5744 CVRSq 0.11126

Note: the cross-validation sd's below are standard deviations across folds

Cross validation: nterms 3.10 sd 0.32 nvars 2.00 sd 0.00

CVRSq	sd	MaxErr	sd
0.111	0.567	-0.116	0.0896

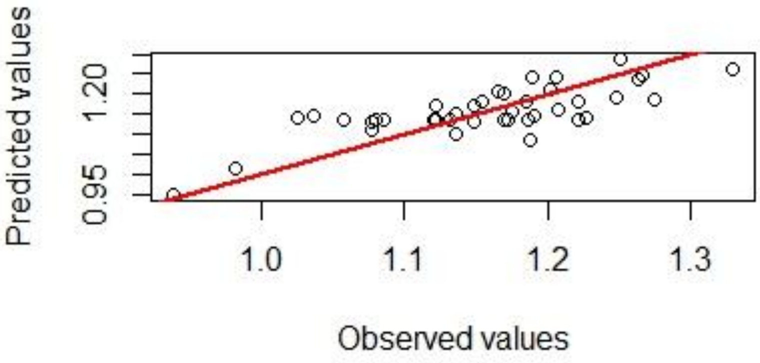

Figure 1.2.1: Observed values plotted against the predicted values of Rc_{2002}.

1.2.2. Using MAT and Summer_SMI as predictors

- Equation retained by the earth model:

Rc_{2002}= 1.1558 - 0.081771 * pmax(0, MAT - 3.9419) + 0.03287 * pmax(0, 93.285 - Summer_SMI)

- Other summary details:

Selected 3 of 9 terms, and 2 of 2 predictors
Termination condition: RSq changed by less than 0.001 at 9 terms
Importance: MAT, Summer_SMI
Number of terms at each degree of interaction: 1 2 (additive model)
GCV 0.0039038 RSS 0.13109 GRSq 0.38724 RSq 0.4984 CVRSq -0.36596

Note: the cross-validation sd's below are standard deviations across folds

Cross validation: nterms 3.00 sd 0.00 nvars 1.90 sd 0.32

CVRSq	sd	MaxErr	sd
-0.366	1.11	-0.205	0.113

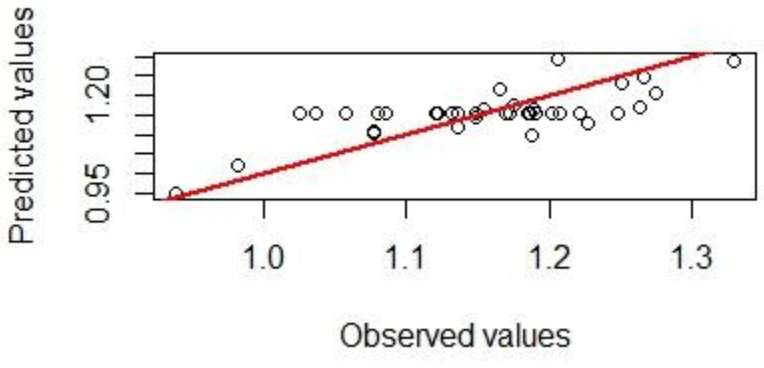

Figure 1.2.2: Observed values plotted against the predicted values of Rc_{2002}.

1.3. Detailed MARS analyses for growth relative resilience, Rr_{2002}

1.3.1. Using MAT, Summer_SMI, ADD and MAP as predictors

- Equation retained by the earth model:

Rr_{2002} = 0.096771- 0.060174 * pmax(0. MAT - 3.9419) + 0.0038915 * pmax(0, 217.94 - ADD)

- Other summary details:

Selected 3 of 15 terms, and 2 of 4 predictors
Termination condition: Reached nk 21
Importance: MAT, ADD, ...
Number of terms at each degree of interaction: 1 2 (additive model)
GCV 0.0016234 RSS 0.054517 GRSq 0.51058 RSq 0.59936 CVRSq -1.2082

Note: the cross-validation sd's below are standard deviations across folds

Cross validation: nterms 3.10 sd 0.57 nvars 1.90 sd 0.32

CVRSq	sd	MaxErr	sd
-1.21	2.16	-0.166	0.0855

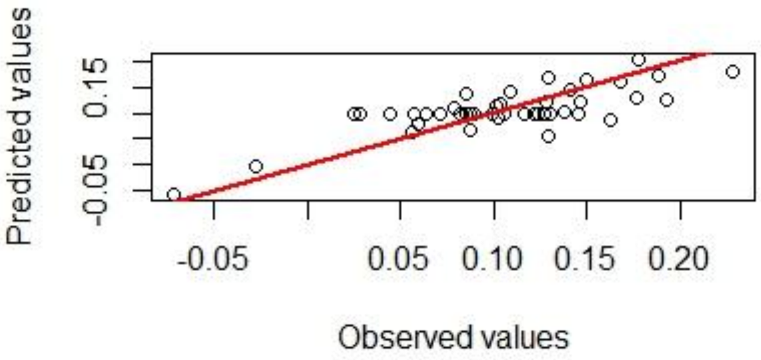

Figure 1.3.1: Observed values plotted against the predicted values of Rr_{2002}.

1.2.2. Using MAT and Summer_SMI as predictors

- Equation retained by the earth model:

Rr_{2002} = 0.10016- 0.061769 * pmax(0. MAT - 3.9419) + 0.019388 * pmax(0, 94.354 - Summer_SMI)

- Other summary details:

Selected 3 of 9 terms, and 2 of 2 predictors
Termination condition: RSq changed by less than 0.001 at 9 terms
Importance: MAT, Summer_SMI
Number of terms at each degree of interaction: 1 2 (additive model)
GCV 0.0016876 RSS 0.056671 GRSq 0.49125 RSq 0.58354 CVRSq -1.9931

Note: the cross-validation sd's below are standard deviations across folds

Cross validation: nterms 3.40 sd 0.52   nvars 2.00 sd 0.00

CVRSq	sd	MaxErr	sd
-1.99	6.57	-0.105	0.0739

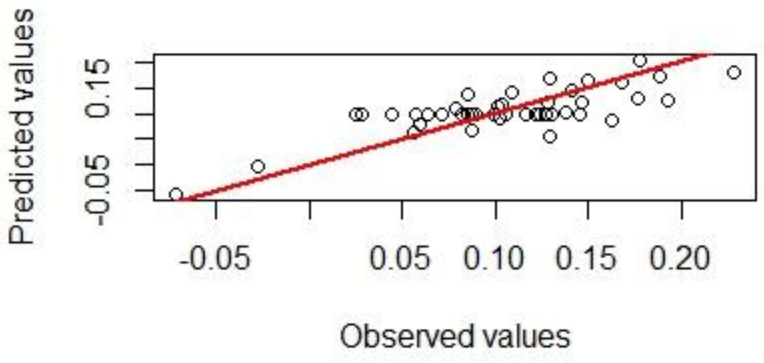

Figure 2.3: Observed values plotted against the predicted values of Rr_{2002}.
